# Transcriptional Portrait of *Actinobacillus pleuropneumoniae* during Acute Disease - Potential Strategies for Survival and Persistence in the Host

**DOI:** 10.1371/journal.pone.0035549

**Published:** 2012-04-17

**Authors:** Kirstine Klitgaard, Carsten Friis, Tim K. Jensen, Øystein Angen, Mette Boye

**Affiliations:** 1 National Veterinary Institute, Technical University of Denmark, Frederiksberg C, Denmark; 2 National Food Institute, Technical University of Denmark, Kongens Lyngby, Denmark; Université Paris Descartes; INSERM, U1002., France

## Abstract

**Background:**

Gene expression profiles of bacteria in their natural hosts can provide novel insight into the host-pathogen interactions and molecular determinants of bacterial infections. In the present study, the transcriptional profile of the porcine lung pathogen *Actinobacillus pleuropneumoniae* was monitored during the acute phase of infection in its natural host.

**Methodology/Principal Findings:**

Bacterial expression profiles of *A. pleuropneumoniae* isolated from lung lesions of 25 infected pigs were compared in samples taken 6, 12, 24 and 48 hours post experimental challenge. Within 6 hours, focal, fibrino hemorrhagic lesions could be observed in the pig lungs, indicating that *A. pleuropneumoniae* had managed to establish itself successfully in the host. We identified 237 differentially regulated genes likely to encode functions required by the bacteria for colonization and survival in the host. This group was dominated by genes involved in various aspects of energy metabolism, especially anaerobic respiration and carbohydrate metabolism. Remodeling of the bacterial envelope and modifications of posttranslational processing of proteins also appeared to be of importance during early infection. The results suggested that *A. pleuropneumoniae* is using various strategies to increase its fitness, such as applying Na+ pumps as an alternative way of gaining energy. Furthermore, the transcriptional data provided potential clues as to how *A. pleuropneumoniae* is able to circumvent host immune factors and survive within the hostile environment of host macrophages. This persistence within macrophages may be related to urease activity, mobilization of various stress responses and active evasion of the host defenses by cell surface sialylation.

**Conclusions/Significance:**

The data presented here highlight the importance of metabolic adjustments to host conditions as virulence factors of infecting microorganisms and help to provide insight into the mechanisms behind the efficient colonization and persistence of *A. pleuropneumoniae* during acute disease.

## Introduction

Due to technical limitations and ethical considerations, most transcriptional studies of pathogenic bacteria have, until recently, been *in vitro* experiments intended to simulate microenvironments of the host in a simplified system. These studies have provided valuable insight into bacterial pathogenesis, but must be interpreted with caution, as the results of *in vitro* models are influenced by the model system used [1]–[3]. Real-life pathogenesis is a multifactorial process where the microbe is challenged by host immune factors and constant changes in nutrient availability [4]. The molecular mechanisms involved in bacterial pathogenicity can only be studied in depth in the natural host, where the gene expression profile accurately depicts the many concurrent responses that reflect the physiochemical conditions of the *in vivo* site of infection [5], [6]. Due to technical improvements, it has now become possible to obtain prokaryotic mRNA from *in vivo* infections of a quality and quantity sufficient to perform whole genome transcriptional analysis [7]–[11].

The objective of this study was to gain a detailed understanding of the molecular basis of pathogenicity in the disease porcine pneumonia, by measuring the host-adapted genomic transcriptional response of *A. pleuropneumoniae* in its natural host during acute infection. This highly infectious respiratory disease is the cause of impaired animal welfare and serious economic losses in swine herds world-wide [12]. The etiological factor, *A. pleuropneumoniae*, is a Gram-negative, facultative anaerobic coccobacillus of the *Pasteurellaceae* family [13]. Macroscopically, the affected lung is characterized by fibrinohemorrahagic necrotizing bronchopneumonia and fibrinous pleuritis [14]. The infection can range from peracute disease with rapid death to chronic infection resulting in asymptomatic carriers [12]. Based on antigenic properties of the capsular polysaccharides and the cell wall lipopolysaccharides *A. pleuropneumoniae* has been divided into 15 serotypes, among which some variance in virulence has been observed [14]. There are three basic stages in the pathogenesis of porcine pneumonia: colonization, subversion of host defense, and damage to host tissue [12]. Some of the virulence factors involved in these stages have been identified, such as adhesins, iron-acquisition factors, capsule and lipopolysaccharides and in particular the RTX toxins, which are major virulence factors of *Pasteurellaceae* [12], [14]. Still, important aspects of fundamental molecular processes in the host-pathogen interactions of this disease remain to be elucidated, e.g. which factors enable the successful survival and persistence of *A. pleuropneumoniae* in the host. Many of the presently known virulence factors have been identified by *in vivo* methods such as signature tagged mutagenesis (STM), *in vivo* expression technology (IVET) and selective capture of transcribed sequences (SCOTS) [15]–[19].

The sequencing of the whole genome of a selection of serotypes of *A. pleuropneumoniae* and the construction of genome-wide microarrays has led to a number of interesting studies on the traits underlying infection, for example during biofilm formation and in environments mimicking the conditions in the lung during early infection [20]–[22]. One of the few presently published genome-wide transcriptional profiling studies of a bacterial pathogen in its natural host was performed on *A. pleuropneumoniae* [11]. Hitherto, both *in vivo* and in *vitro* studies of bacterial genomic expression have included only a few samples. Here we present what is, to the best of our knowledge, the first large scale time-course *in vivo* transcriptome study of a bacterium in its natural host. We compared expression profiles of *A. pleuropneumoniae* recovered from the lungs of 25 pigs at four time-points during the first 48 hours after experimental challenge. In this study we gained important information of the bacterial strategy during establishment and survival in the host and identified putative new virulence factors.

## Results

### *In vivo* transcriptome approach

We used a custom designed *A. pleuropneumoniae* NimbleGen microarray to characterize the transcriptional profile of *A. pleuropneumoniae* serotype 2 and serotype 6 in a time study 6, 12, 24 and 48 hours post infection (p.i.). Serotypes 2 and 6 represent more than 90% of the clinical isolates originating from Denmark. Visual macroscopic infection was confirmed established in 28 of the 48 experimentally infected pigs. Details of the sampled material are listed in Table S1. Cultivation from infected lungs revealed that, except for 3 animals, co-infection mainly by *Pasteurella multocida* but also *Streptococcus suis* and non-hemolytic *Escherichia coli*, could be observed (Table S1). Most likely, these bacteria were present before the inoculation with *A. pleuropneumoniae*. As the array was designed to be highly specific for *A. pleuropneumoniae*, comprising many short oligonucleotides for each gene (covered by an average of 26.7 probes of a mean size of 48 bp), cross-hybridization of other bacteria than *A. pleuropneumoniae* to the microarrays was expected to be minimal. This assumption was supported by a Pearson's correlation coefficient of 0.93 between the pure cultures of *A. pleuropneumoniae* and the mixed bacterial cultures; calculated from the median expression values of each gene within the pure culture arrays versus the mixed culture arrays.

Total RNA was extracted from three lung samples of each infected animal (n = 84). The mRNA was linearly amplified to obtain sufficient material for microarray analysis. To test whether the data had been skewed by the amplification procedure, the expression of 11 bacterial genes before and after mRNA amplification was validated by quantitative real-time RT-PCR (qPCR). Samples included three individual RNA extractions from infected lung tissue of pig no. 33 and 55, before and after linear amplification, respectively (n = 6). The results of the qPCR analysis showed good correlation between expression of the selected genes before and after amplification (Spearman's rho 0.74, P<0.009 for both animals) (Table S2).

**Figure 1:**
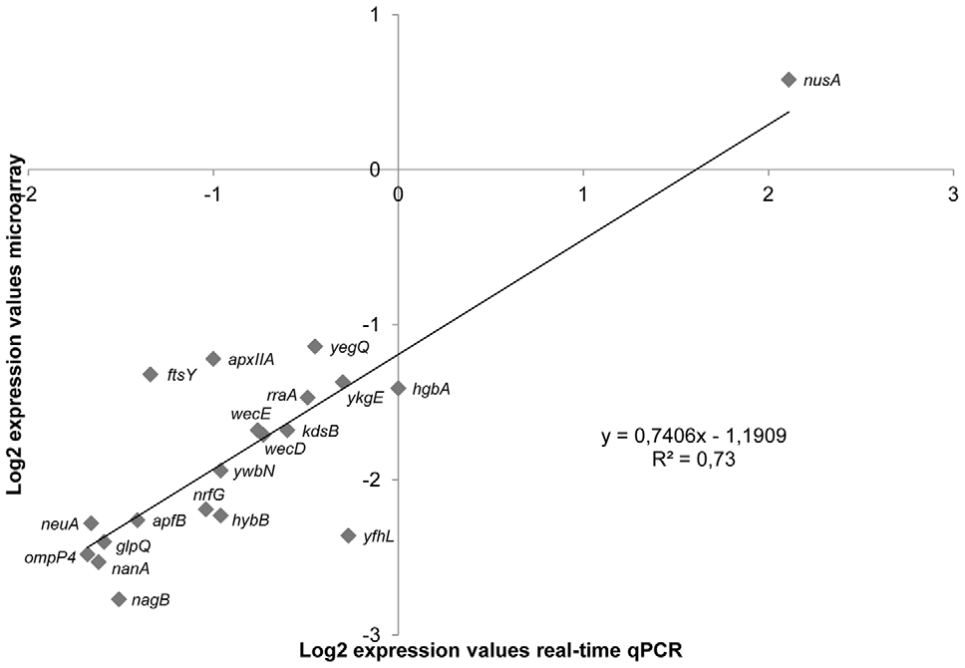
Validation of microarray results by qPCR. Microarray and qPCR analyses were applied to measure *in vivo* expression changes between 6 h p.i. and 48 h p.i. for 20 selected *A. pleuropneumoniae* genes. The log_2_ transformed microarray data were plotted against the log_2_ transformed qPCR data for correlation analysis.

Microarrays from three animals (triplicates; n = 9) were discarded due to lack of sufficient signal detection, leaving 75 microarrays from 25 pigs for further downstream analysis. A density plot of the 75 normalized microarrays is depicted in Figure S1 and reveals a clear distinction between the background and the expression signal. The reliability of the microarray data was assessed by qPCR analysis. We selected a subset of 20 genes and compared the results of the qPCR on cDNA from bacteria isolated 6 h (pigs no. 33, 36, 55 and 59) and 48 h (pigs no. 51, 54, 75 and 76) p.i., respectively. Of the 8 pigs, three biological samples (independent mRNA extraction and cDNA synthesis) were included in the analysis (n = 24). The qPCR results and microarray data exhibited a high correlation coefficient (R^2^ = 0.73) (Figure 1).

Genes differentially expressed during acute infection were identified by comparing the whole genome transcriptional profiles of bacteria recovered from infected lung tissue at the four time points of infection. This procedure was chosen to avoid introducing noise by comparing the *in vivo* data to an *in vitro* grown bacterial control culture. By two-way ANOVA analysis using the software R (http://www.r-project.org/), 250 open reading frames (ORFs) were identified as significantly (P<2×10^−9^) differentially expressed during the first 48 h of infection. These 250 open reading frames corresponded to 237 unique genes (Table S3). With very few exceptions, most of these genes displayed a steady decline in expression from 6 h to 48 h p.i.. It is reasonable to assume that the observed changes in gene expression during the first 48 h of infection were related to the changes induced by the bacteria entering the host; but without an *in vivo* expression value for time zero of the infection (not obtainable due to technical limitations), we cannot substantiate this hypothesis. It has previously been shown, however, that bacterial gene expression in response to environmental changes happens very rapidly and mainly through gene activation [5], [23]. A possible explanation for the observed differential expression, where genes are induced early and then gradually decline in expression, is that it may reflect the gradual adaption of *A. pleuropneumoniae* to the new environmental conditions; possibly characterized by the gradual deterioration of the host.

Because no suitable reference could be established for this experimental set-up, important virulence genes might be overlooked if these were constitutively expressed during infection. We therefore included the constitutively most highly expressed genes in the analysis. To avoid problems with background noise and to keep the number of genes under investigation at a manageable size, we selected a cut off value of mean log_2_≥13 (SD<0.5), resulting in 133 ORFs which were the constitutively most highly expressed genes (Table S4).

**Figure 2:**
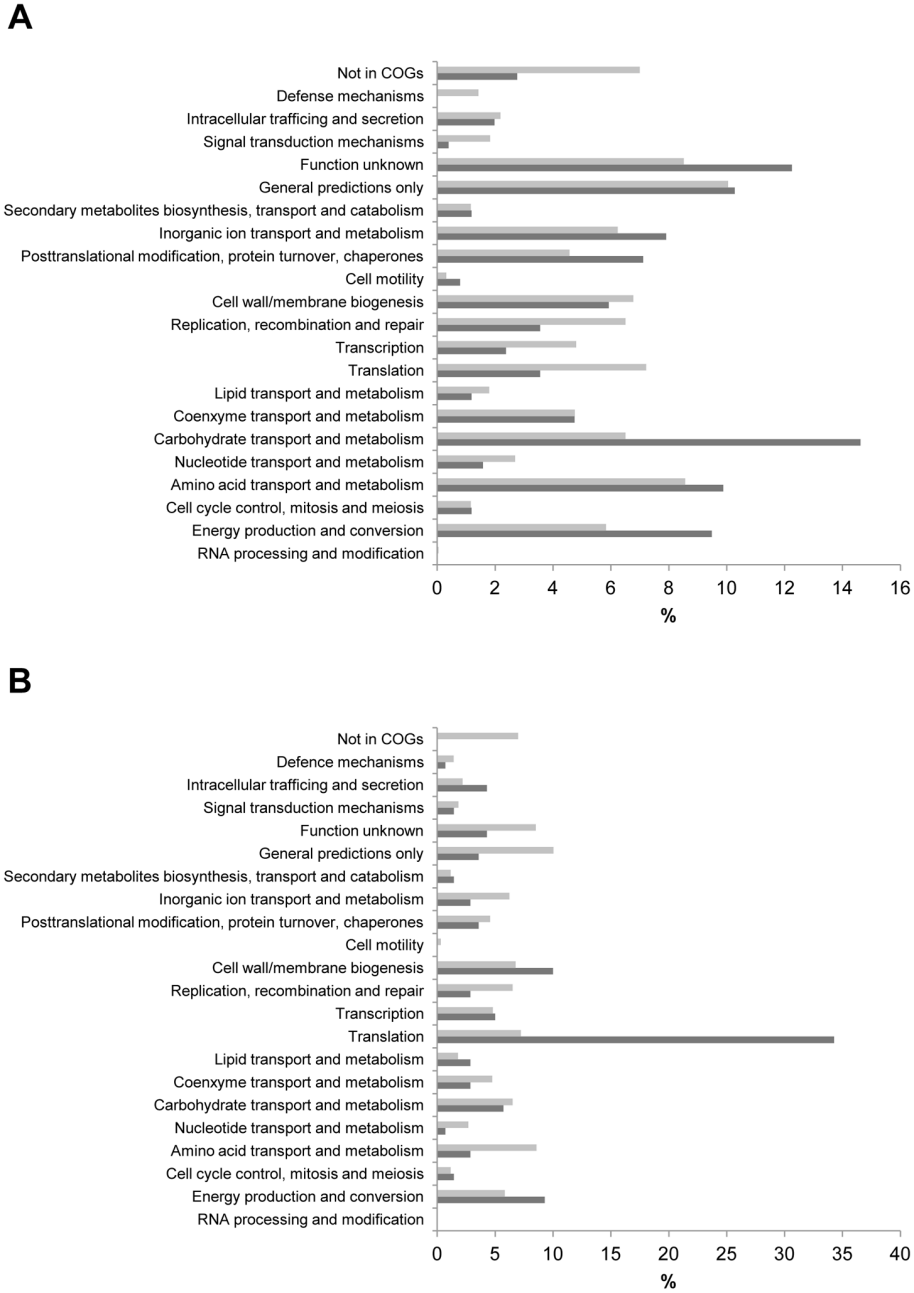
Distribution of *in vivo* regulated genes classified according to the Clusters of Orthologous groups (COGs). Dark bars: Functional distribution of differentially expressed genes (A) and constitutively highly expressed genes (B) in *A. pleuropneumoniae* during growth in pig lung in the acute phase of infection. Light bars: distribution of functional groups in the *A. pleuropneumoniae* genome.

On the basis of clusters of orthologous groups (COGs) classifications, categories that were overrepresented in the differentially expressed gene set relative to their representation in the *A. pleuropneumoniae* genome overall [24], were “energy production and conversion”, “carbohydrate transport and metabolism”, “post translational modification, protein turnover and chaperones”, “amino acid transport and metabolism”, “inorganic ion transport and metabolism”, “cell motility” and “unknown functions” (Figure 2A). Ribosomal proteins or those involved in translation, and to a lesser degree transcription, dominated the group of constitutively highly expressed genes, both with regards to numbers of genes (31.5%) and level of expression (between log_2_ of 13.9 and 15.2). The high expression of ribosomal genes indicated a high growth rate *in vivo* (Figure 2B). Likewise overrepresented among the constitutively highly regulated genes were the functional groups “cell wall/membrane biogenesis” and “intracellular trafficking and secretion” (Figure 2B).

### Comparison with other expression studies

Although direct comparison was complicated by differences in experimental designs, we cross-referenced our findings with recent expression studies of *A. pleuropneumoniae* [11], [16]–[18], [20]–[22] and another member of the *Pasteurellaceae* family, *Haemophilus influenzae* [25] (Table S5). Around 38% of the differentially regulated genes and 43% of the constitutively highly expressed genes, identified in the present study, had previously been identified as being differentially regulated during the infectious process, or during biofilm formation.

### Adhesion and competence

*A. pleuropneumoniae* enters the airways after inhalation as an aerosol and colonizes the host by binding to mucus, proteins and host cells in the distal parts of the lung. This ability to adhere to host cells or surfaces is a vital part of a successful bacterial invasion [12], [14]. Not surprisingly, we saw differential regulation of the type IV pilus genes (*apfAB*), most likely induced by contact with lower respiratory tract epithelial cells thereby promoting adherence to these cells [11], [26]. Two of the constitutively highly expressed genes, *csgG* and *tufB*, were also potential participants in the adhesion process. *CsgG* encodes a component in the production of long thin aggregative fimbriae (curli) with adhesive properties [27]. *CsgG* had previously been observed to be up-regulated *in vivo* in pig lung during the acute phase of disease and during biofilm formation [11], [22]. Interestingly, the curli protein assembly was identified as a potent immunogenic protein in *Haemophilus parasuis*, the cause of Glässer's disease in pigs [28]. The elongation factor *tufB*, has also previously been identified as a potential virulence factor, and is possibly involved in fibronectin binding [18]. Although the competence gene, *comE1*, has been suspected to be involved in adhesion [29], *in vivo* regulation of *comE1* has not previously been reported. Also not previously described in *A. pleuropneumoniae* was the observed *in vivo* differential transcription of at least six genes with putative involvement in competence. Besides *comE1* and *apfAB*, these were: *comB*, *hofQ*, *comM* and *radC* [30].

### Metabolic adaptations to *in vivo* conditions in the porcine lung

The results indicated that at 6 h p.i. *A. pleuropneumoniae* were encountering anaerobic conditions in the porcine lung. Table 1 lists the 32 genes involved in anaerobic metabolism that were displaying variations in gene expression during acute infection. Differential expression of the reductases, *torYZ*, *dmsA* and *nrfABCEFG*, suggested that *A. pleuropneumoniae* was using trimethylamine oxide, dimethyl sulfoxide and nitrite as terminal electron acceptors in anaerobic respiration [31]. *DmsA* has previously been demonstrated to have an effect on *A. pleuropneumoniae* virulence [32]. Among the significantly regulated genes were also the Ni/Fe cofactor dependent hydrogenases (*hyaABD*; *hybAB*), which catalyze the production and consumption of hydrogen gas.

**Table 1:** Differentially expressed *A. pleuropneumoniae* genes involved in anaerobic metabolism.

Gene designation	Locus no.^a^	Annotation	Functional group^b^	Log_2_ mean expression 6 h (n = 21)	Log_2_ mean expression 12 h (n = 15)	Log_2_ mean expression 24 h (n = 18)	Log_2_ mean expression 48 h (n = 21)	P-value (differential expression)
*nrfC* *	APL_0102	Nitrate reductase	C	12.41	12.47	11.19	10.92	1.20E-09
*glpA*	APL_0379	Sn-glycerol-3-phosphate dehydrogenase subunit A	C	11.18	10.93	10.48	9.94	1.96E-13
*torZ* *	APL_0688	Trimethylamine-N-oxide reductase precursor	C	12.73	12.13	11.31	10.54	1.21E-16
*torY*	APL_0689	Cytochrome c-type protein	C	12.66	12.13	11.23	10.44	2.82E-15
*dcuC* *	APL_0870	Putative C4-dicarboxylate transporter	C	11.21	11.04	10.12	9.46	6.39E-14
*hyaA* *	APL_1331	Hydrogenase 2 small subunit	C	10.03	9.15	8.3	7.82	2.55E-22
*hybA*	APL_1332	Hydrogenase 2 protein	C	11	10.26	9.26	8.81	1.75E-21
*hybB* *	APL_1333	Putative Ni/Fe-hydrogenase 2 b-type cytochrome subunit	C	12.03	11.64	10.39	9.72	6.76E-14
*hyaB* *	APL_1334	Hydrogenase-2 large chain	C	12.24	11.69	10.4	9.94	2.46E-15
*hyaD*	APL_1335	Hydrogenase 2 maturation protease	C	12.46	11.95	11.18	10.8	3.99E-18
*oadA*	APL_1376	Oxaloacetate decarboxylase alpha chain	C	11.25	11.1	10.17	9.78	1.59E-09
*oadB*	APL_1377	Oxaloacetate decarboxylase beta chain	C	11.45	11.42	10.54	10.01	1.46E-09
*dmsA* *	APL_1674	Anaerobic dimethyl sulfoxide reductase chain A precursor	C	13.17	13.32	12.3	11.7	1.34E-09
*dhaM*	APL_0081	PTS-dependent dihydroxyacetone kinase. phosphotransferase subunit	S	11.49	11.14	10.31	9.94	1.07E-14
*dhaL*	APL_0082	PTS-dependent dihydroxyacetone kinase. ADP-binding subunit	G	12.55	12.3	10.86	10.42	4.31E-14
*dhaK*	APL_0083	PTS-dependent dihydroxyacetone kinase. dihydroxyacetone-binding subunit	G	11.37	11.1	9.94	9.08	1.29E-17
*malK* *	APL_1236	Maltose/maltodextrin import ATP-binding protein	G	10.8	10.61	9.42	8.72	1.08E-11
*malG* *	APL_1239	Maltose transport system permease protein	G	9.88	9.53	8.83	8.04	3.59E-13
*malQ* *	APL_1240	4-alpha-glucanotransferase	G	12.57	11.99	11.16	10.62	1.22E-09
*ulaD* *	APL_1698	Probable 3-keto-L-gulonate-6-phosphate decarboxylase	G	11.92	10.91	10.38	10.27	1.05E-10
*ulaC* *	APL_1699	Ascorbate-specific phosphotransferase enzyme IIA component	G	9.83	9.02	8.88	8.74	9.09E-14
*ulaG* *	APL_1701	L-ascorbate-6-phosphate lactonase UlaG-like protein	R	12.45	11.92	11.37	11.03	9.03E-11
*ulaR*	APL_1702	HTH-type transcriptional regulator	G	11.3	10.74	9.68	9.36	4.27E-13
*ulaA*	APL_1714	Ascorbate-specific permease IIC component	S	11.35	10.67	9.57	8.48	6.02E-18
*chuW* *	APL_1523	Coproporphyrinogen III oxidase	H	11.28	10.7	9.86	9.36	1.45E-13
*nrfG*	APJL_1067	Formate-dependent nitrite reductase complex	O	10.98	10.36	9.35	8.79	4.79E-14
*nrfF*	APJL_1068	Formate-dependent nitrite reductase complex	O	9.39	9.08	8.66	8.33	8.67E-13
*nrfE*	APL_1052	Cytochrome c-type biogenesis protein	O	11.23	11.2	10.43	9.93	2.94E-10
*nrfA*	APL_0100	Cytochrome c-552	P	13.64	13.56	12.22	12	1.10E-10
*nrfB* *	APL_0101	Cytochrome c-type protein	P	12.54	12.4	10.81	10.43	2.60E-10
*dcuB2* *	APL_1316	Anaerobic C4-dicarboxylate transporter	R	12.63	12.44	11.72	11.29	1.31E-11
*dcuB1*	APL_1532	Anaerobic C4-dicarboxylate transporter	R	12.99	12.82	11.95	11.67	1.08E-12

* Genes previously identified as being relevant for *A. pleuropneumoniae* infection [11], [18], [20], [21].

a Locus numbers from *A. pleuropneumoniae* serotype 5 (L20) or alternatively, if gene could not be identified in serotype 5, then from *A. pleuropneumoniae* serotype 3.

b Function of genes according to Clusters of Clusters of Orthologous Groups of proteins (COGs). C: energy production and conversion; G: carbohydrate transport and metabolism; H: coenzyme transport and metabolism; O: posttranslational modification, protein turnover, chaperones; P: inorganic ion transport and metabolism; R: general function prediction, only; S: function unknown.

Additionally, genes of the Na+ pump, oxaloacetate decarboxylase (*oadAB*) were differentially regulated over time while the Na+-exporting NADH dehydrogenase (*nqrABCEF*) genes were constantly highly up-regulated.

The largest functional group of differentially regulated genes (14.5%) (Figure 2A) was predicted to be involved in carbohydrate transport and metabolism, which indicated a major shift in utilization of carbon sources for *A. pleuropneumoniae* when colonizing the host. Our results agreed with previous observations that genes responsible for transport and anaerobic metabolism of maltose (*mal*) and ascorbate (*ula*), respectively, were important in the acute phase of infection [11]. But, in addition, genes involved in uptake and metabolism of xylose (*xylAFGH*) and galactose (*galK, mglABC*) exhibited differential regulation over time. Also, two anaerobic pathways for glycerol dissemination (*glp* and *dha*) were significantly regulated *in vivo*. Both leads to the formation of dihydroxyacetone phosphate (DHAP), an intermediate of glycolysis [33]. Glycerol is an essential precursor for the synthesis of lipids and seems to be an important carbon/energy source for pathogenic bacteria [33].

The differential expression of methylglyoxal synthase, encoded by the *mgsA* gene, could be a sign that *A. pleuropneumoniae* is actually experiencing carbon excess in early phase of infection. This bypass system produces methylglyoxal (MG) from the excess supply of DHAP [34], [35]. MG is an extremely toxic electrophile, and the bacteria must therefore, rather paradoxical, protect itself against its own product. In *E. coli*, the principal route of MG detoxification is the glutathione-dependent glyoxalase system consisting of two enzymes glxI (*gloA*) and glxII (*gloB*) [34], [36]. The glutathione conjugates activate the potassium efflux genes *kefB* and *kefC* which leads to a lowering of the intracellular pH of the bacterial cell and protection against the toxic effects of MG [34]. While *gloA* and *gloB* appeared to be active at the same level throughout the trial, the *kefB* and *kefC* genes were differentially expressed during infection. *KefB* and *kefC* have also earlier been found regulated in *A. pleuropneumoniae* isolated from necrotic porcine lung tissue [18].

Few *in vivo* regulated genes involved in iron acquisition were found. We observed differential regulation of *fhuC*, which is part of an operon encoding proteins involved in uptake of exogenously, supplied siderophores [37] and *afuAB*, constituting a periplasmic protein-dependent ABC-type Fe^3+^ transport system [38]. For microorganisms trying to colonize the mucosal surfaces of their host, haemin is a potentially valuable source of iron [39] and we also recorded differential regulation of *hmuV*, encoding a possible hemin ABC superfamily ATP binding cassette transporter. This gene has been reported not to be present in the commensal strains of *Pasteurellaceae* [40].

### Cell wall metabolism

Synthesis of products related to cell wall/membrane biogenesis appears to be in high demand in early infection as this functional group was overrepresented among constitutively highly regulated genes (Figure 2B). Table 2 summarizes the 32 differentially or constitutively highly expressed genes with putative or known functions in cell wall/membrane biogenesis. A key enzyme in the biosynthesis of lipopolysaccharide (LPS), CMP-Kdo synthetase (*kdsB*), was differentially regulated over time. The LPS are some of the major surface components that interact directly with factors in the host environment and play a vital role in the infectious process [41] and the *kdsB* enzyme may constitute a possible target for the development of new antimicrobial agents against Gram-negative bacteria [42].

**Table 2:** Differentially or highly expressed *A. pleuropneumoniae* genes with expected or putative functions in cell wall/membrane biogenesis.

Gene designation	Locus no.^a^	Annotation	Functional group^b^	Log_2_ mean expression 6 h (n = 21)	Log_2_ mean expression 12 h (n = 15)	Log_2_ mean expression 24 h (n = 18)	Log_2_ mean expression 48 h (n = 21)	P-value^c^ (differential expression)
*rsmH*	APL_0010	Ribosomal RNA small subunit methyltransferase H	M	11.39	11.18	10.71	10.45	1.30E-11
*murD* *	APL_0016	UDP-N-acetylmuramoylalanine-D-glutamate ligase	M	11.63	11.32	10.69	10.35	3.26E-11
*ftsQ*	APL_0021	Cell division protein	M	13.23	13.40	12.96	13.00	NS
*lpxC*	APL_0024	UDP-3-O-[3-hydroxymyristoyl] N-acetylglucosamine deacetylase	M	13.41	13.36	13.02	13.09	NS
*kdsB*	APL_0085	3-deoxy-manno-octulosonate cytidylyltransferase	M	10.82	10.27	9.50	9.14	8.10E-10
*prc* *	APL_0120	Carboxy-terminal protease	M	13.21	13.29	12.97	13.09	NS
*csgG* *	APL_0220	Putative lipoprotein	M	13.91	14.07	14.05	14.18	NS
*APL_0221*	APL_0221	Putative lipoprotein. periplasmic protein	S	14.35	14.46	14.46	14.58	NS
*APL_0234*	APL_0234	23S rRNA pseudouridine synthase D	M	11.92	11.36	10.55	10.33	1.53E-11
*tolA*	APL_0302	Cell envelope integrity inner membrane protein	M	13.24	13.27	13.07	13.44	NS
*palA*	APL_0304	Outer membrane protein PalA	M	14.51	14.41	14.17	14.15	NS
*nlpC* *	APL_0359	Putative lipoprotein	M	13.31	13.41	13.20	13.20	NS
*ompP4* **	APL_0389	Lipoprotein E	R	12.46	11.71	10.77	9.98	4.29E-14
*macA*	APL_0391	Probable macrolide-specific efflux protein	M	11.74	11.44	10.52	10.49	4.45E-10
*nlpI*	APL_0576	Lipoprotein NlpI-like	R	13.89	14.06	14.06	14.15	NS
*acrA*	APL_0586	Putative RND efflux membrane fusion protein	M	13.27	13.43	13.32	13.45	NS
*dgkA*	APL_0768	Diacylglycerol kinase	M	11.38	10.90	10.33	10.04	3.99E-11
*lrgB*	APL_0779	Putative effector of murein hydrolase	M	10.63	9.83	9.16	8.40	7.16E-15
*mltA*	APL_0816	Murein transglycosylase A	M	13.19	13.31	13.15	13.26	NS
*ompW* *	APL_1086	Outer membrane protein W	M	12.07	11.88	10.80	10.17	7.01E-12
*APL_1121*	APL_1121	Putative lipoprotein	R	13.70	13.66	13.38	13.39	NS
*ompA* *	APL_1421	Outer membrane protein P5 precursor	M	14.18	14.18	14.02	13.92	NS
*wecE*	APL_1549	TDP-4-keto-6-deoxy-D-glucose transaminase	M	10.13	9.71	8.98	8.45	1.07E-11
*wecD*	APL_1550	Putative TDP-D-fucosamine acetyltransferase	M	9.71	9.05	8.58	8.00	6.51E-16
*wecC*	APL_1551	UDP-N-acetyl-D-mannosamine dehydrogenase	M	10.45	10.03	9.33	8.69	8.68E-15
*wecB*	APL_1552	UDP-N-acetylglucosamine 2-epimerase	M	10.63	10.28	9.37	8.91	1.33E-11
*APL_1597* *	APL_1597	Rare lipoprotein A	M	13.93	13.80	13.40	13.39	NS
*glmS*	APL_1631	Glucosamine–fructose-6-phosphate aminotransferase	M	12.74	13.12	13.11	13.19	NS
*mltC* **	APL_1741	Membrane-bound lytic murein transglycosylase C	M	13.27	13.23	12.93	12.85	NS
*murl* *	APL_1841	Glutamate racemase	M	11.08	10.65	10.13	9.77	9.54E-13
*ompA*	APL_1852	Outer membrane protein P5 precursor (OMP P5)	M	14.55	14.59	14.50	14.50	NS

* Genes previously identified as being relevant for *A. pleuropneumoniae* infection or biofilm formation [11], [17], [18], [20]–[22].

** *Haemophilus influenzae* genes required in the lung determined in a murine pulmonary model of infection [25].

a Locus numbers from *A. pleuropneumoniae* serotype 5 (L20).

b Function of genes according to Clusters of Clusters of Orthologous Groups of proteins (COGs). M: cell wall/membrane biogenesis; R: general function prediction, only; S: function unknown.

c P-values are only included for the genes that were differentially expressed over time, the constitutively highly expressed genes were of course not significant (NS).

Likewise, genes from the peptidoglycan biosynthetic pathway (*murD* and *murI*) were differentially expressed in the initial stages of infection, along with *lrgB*, a putative effector of murein hydrolase. Murein hydrolases are needed in order to expand the cell wall during bacterial growth and may therefore be important factors in determining the course of infection [43].

The enterobacterial common antigen (ECA) is a glycolipid present in the outer membrane in Gram-negative enteric bacteria. Genes responsible for the biosynthesis of ECA, (*wecBCDE*) were differentially expressed in this study. Probably belonging to the same operon and also differentially expressed over time was the O-antigen translocase, *wzxE*. The genes *wecABCG* are required for synthesis of lipid I and lipid II, while *wecE* is involved in lipid III synthesis. Lipid III is transported across the membrane via the *wxz* translocase [44]. We were not able to find other studies describing *in vivo* or *in vitro* up-regulation of ECA in *A. pleuropneumoniae*. Although present in all Gram-negative enteric bacteria, the function of ECA remains to be established.

It has previously been demonstrated that both lipoprotein E (*ompP4*) and the outer membrane protein P5 (*ompP5*/*ompA*) play active parts in the pathogenesis of *H. influenzae* [45], [46]. In the current study, ompP4 was differentially regulated while the two ompP5 genes (*APL_1421* and *APL_1852*), were constitutively highly expressed.

### Stress response

The classical chaperones, HSP70 (*dnaK*), HSP40 (*dnaJ*) and *dijA*, and the periplasmic stress sensor, *degS*, were found to be constitutively highly expressed and may be important for bacterial survival within macrophages. Hsp70 and its co-chaperones are the most potent cellular defenses against environmental insults [47]. *DnaK* was reported as immunoreactive in convalescent sera from pigs naturally infected with *A. pleuropneumoniae* [48]. We observed *in vivo* activation of oxidative stress resistance mechanisms, represented by the two genes coding for thiol peroxidase (*tpx*) and cytochrome c peroxidase (*ccp*), respectively, both involved in protecting the bacteria against hydrogen peroxide [49]. In *A. pleuropneumoniae* grown in bronchoalveolar fluid, *ccp* has earlier been found to be among the most highly up-regulated genes [21]. Also, it has been reported that the lipid hydroperoxide peroxidase, encoded by *tpx*, protects *Salmonella enterica* from hydrogen peroxide stress in vitro and facilitates intracellular growth [50].

In the lungs, copper concentrations have been shown to increase during infection and inflammation [51]. As high concentrations of copper are toxic, bacteria have developed a number of mechanisms for dealing with excess concentrations of this metal. Efflux mechanisms include the ubiquitous *copA*/*copB* P1-type ATPase transporters [51]. We noticed significant regulation of *copA* (APL_1265) and the putative cation transport ATPase, most likely involved in copper detoxification (APL_1264). *CopA* has earlier been found to be important for survival in necrotic porcine lung tissue [18].

Urease activity may increase intracellular survival and impair macrophage function through the production of ammonia, which inhibits phagosome-lysosome fusion in macrophages [12], [52], [53]. The genes *ureAGE* were up-regulated in *A. pleuropneumoniae* during biofilm formation *in vitro* [22], but to our knowledge, this is the first report of significant regulation of urease genes (*ureADEG*) during *in vivo* infection. We did not, however, observe any differential regulation of the putative nickel and cobalt periplasmic permease system (*cbiKLMQO*) upstream of the urease cluster which appears to be required for urease activity in this bacterium [54]. As more than one of the biological systems, which requires Ni^2+^ for activity, were significantly regulated in this study (urease, NiFe hydrogenases), it is unclear why no nickel transport proteins appear to be regulated *in vivo*. *A. pleuropneumoniae* may harbor mechanisms of nickel uptake that have yet to be identified, similar to many other bacteria which use nickel without possessing homologues of the known nickel/cobalt transporters [55].

### Evading host immune response

Our results indicated that sialic acid metabolism could be of importance for the survival and persistence of *A. pleuropneumoniae* in the porcine lung. Sialic acids are the terminal sugars of the host cellular glycocalyx and therefore one of the first substances that the microbe encounters when it enters the host [56]. Sialic acid is an attractive nutritional source for microbes that associate with vertebrates [56]. In this study, five putatively co-regulated genes related to sialic acid metabolism, were differentially regulated. These were *neuA*, *nanEA* and *nagBA*. Activation of the *nan* operon depends on the availability of sialic acid in the environment. *NanA* cleaves sialic acid to produce N-Acetyl-D-mannosamine and pyruvate. N-Acetyl-D-mannosamine is converted to fructose-6-P and glucosamine-6-P by the concerted action of *nanEK* and *nagAB* [56].

### Intracellular trafficking and secretion

The general secretion pathway (sec-pathway) is the major route of protein translocation across the cytoplasmic membrane in bacteria [57]. We observed constitutively high *in vivo* expression of *secY*, *secB, secD*, *secF* and *yajC*—all genes encoding components of the predicted sec translocation system. Also genes from the sec independent twin-arginine translocation (Tat) export pathway were differentially (*tatA*) or constitutively highly (*tatB*) expressed in this investigation. The Tat pathway is utilized by bacteria to export pre-folded proteins, in particular cofactor containing redox enzymes, across the bacterial inner membrane into the periplasmic compartment [58]. In *H. influenzae*, the *tatA* and *tatB* genes were both earlier found to be among the genes imperative for bacterial survival in a murine lung model [25]. In *E. coli*, Tat deficient mutants displayed phenotypic characteristics consistent with an outer membrane defect [59]. Proteins targeted for export by the Tat pathway usually possess a twin arginine signal motif, ([S/T]RRXFLK) in the N-terminus [60]. In the present study, the Tat signal motif was identified in 7 differentially regulated *A. pleuropneumoniae* genes (*nrfC*, *torZ*, *hyaA*, *hybA, dmsA*, *cpdB* and *ywbN*) by the signal prediction server TatFind (http://signalfind.org/tatfind.html) [61]. Five of these genes, *nrfC*, *torZ*, *hyaA*, *hybA* and *dmsA*, encode proteins belonging to the energy production and conversion functional group and are involved in anaerobic growth.

### *In vivo* expression of exotoxins

It is well established that the secreted pore-forming RTX exotoxins are among the most important virulence factors in *A. pleuropneumoniae*, directly involved in causing necrotic lesions of the target organs [62]. *A. pleuropneumoniae* serotype 2 and 6 secrete the exotoxins *apxII*, *apxIII* and *apxVI*. In this study, only *apxIIIA* (log_2_ = 13.94; SD = 0.34) was above the set threshold for constitutively highly expressed genes. The toxin genes *apxIIA* (log_2_ = 12.94; SD = 0.85) and *apxIVA* (log_2_ = 11.14; SD = 0.30) were, however, both actively transcribed during the study period. The RTX toxin, ApxIV, is not expressed *in vitro* but activates high levels of serum antibodies during infection [63], [64]. The *apxIVA* gene and has previously been demonstrated to be specifically induced during infection [11].

### Global regulation in *A. pleuropneumoniae*

As the only global regulator, the ferric-uptake regulator protein (Fur) was constitutively highly expressed throughout the experiment (Mean log2 = 13.40; SD = 0.46). Fur plays a key role in controlling iron homeostasis at the level of transcription by sensing intracellular iron levels and adjusting gene expression accordingly [65], [66]. This protein directly regulates most iron-acquisition genes in a negative fashion by blocking their transcription when intracellular iron is at an acceptable level. It has now become clear that Fur, through a small (s)RNA named RyhB, also indirectly acts as a transcriptional activator switching on genes, many of which encode iron-rich respiratory complexes [67]. In *E. coli*, a large group of energy metabolism genes was found to be iron and Fur induced, including genes involved in oxidative stress response and virulence [65], [68]. The general pattern of gene expression observed in this study was in accordance with a Fur regulator with ferrous iron bound. Firstly, only a few iron acquisition genes were differentially expressed, which indicated that *A. pleuropneumoniae* was not encountering iron-restriction during this period of the *in vivo* infection. Secondly, a number of genes coding for metabolic enzymes dependent on Fe-S clusters or other iron cofactors (e.g. *dmsA*, *hyaABD* and *torYZ*) appeared to be actively transcribed and may have been indirectly activated by Fur through RyhB [67].

## Discussion

Monitoring bacterial expression *in situ* during infection is an opportunity to gain unique insight into the molecular mechanisms of host-pathogen interactions, as the *in vivo* expression profile of the microbial invader can also serve as indicator of the host microenvironment. To study this process closer in the porcine pathogen *A. pleuropneumoniae*, we undertook the first large scale time-course study of this bacterium's *in vivo* transcriptome during the first 48 h of infection. *A. pleuropneumoniae* was able to establish severe infection in the lungs of the porcine host within 6 h. However, 17 of the 48 pigs did not develop infection, three pigs died before tissue sampling and three samples were discarded during analysis. This resulted in the loss of balance for the data and presents a two-fold challenge in the interpretation. First, the final 9 and 16 samples of serotype 6 and 2, respectively, were biased in that serotype 2 samples were more prevalent, particularly for the 6 h time point. Second, as a consequence of the first, time and serotype could no longer be assumed to be independent. For these reasons, we only considered the time factor and abstained from drawing any conclusions based on serotype. The inclusion of the serotype factor in the 2-way ANOVA should thus be seen as an endeavor to reduce the potential bias this factor might otherwise introduce to the analysis.

From the 25 samples under analysis, many of the differentially regulated genes were not highly expressed at any time point during the monitored period. The differences in expression were, nevertheless, highly significant, which strongly indicated that they may represent factors required by *A. pleuropneumoniae* for the disease process, even if they are not required at great abundance. We are convinced that by identifying genes that are differentially expressed *in vivo* in response to changes in environmental parameters rather than applying an *in vitro* grown culture as reference condition, subtle changes in gene expression were detected that would otherwise have been missed. For example, we were able to see a distinct shift to anaerobic metabolism (Table 1) which was not detected, when comparing *in vivo* results to *in vitro* culture conditions [11]. The data included many genes previously reported to be implicated in virulence of *A. pleuropneumoniae* and other pathogenic Gram-negative bacteria. But in addition to adding further weight to previous observations of *A. pleuropneumoniae* pathogenesis, this investigation also revealed potential new strategies for adapting to the host environment.

### Cross-references to other expression studies

The results most similar to our investigation were obtained from a gene expression analysis of *A. pleuropneumoniae* (serotype 5b, strain L20), isolated from one naturally infected pig during the exponential phase of infection [11]. In this study, 150 differentially expressed genes were identified *in vivo*, when compared to exponentially growing planktonic cultures in rich laboratory media [11]. Of these 150 genes, 38 were also detected in our study (24 differentially expressed/14 constitutively highly expressed). The genes we observed to be differentially regulated were up-regulated in the study of *A. pleuropneumoniae* serotype 5b; while the genes we identified as constitutively highly expressed were mostly down-regulated in that study (Table S5) [11]. Most likely these discrepancies reflected the difficulties of comparing studies with different experimental design; in this case, a study measuring differences between two growth conditions in *A. pleuropneumoniae* serotype 5b [11] versus the present study measuring changes in bacterial response over time in *A. pleuropneumoniae* serotype 2 and serotype 6.

Also included in the comparison, were microarray gene expression profiles of *A. pleuropneumoniae* exposed to bronchoalvolar fluid, attached to lung epithelial cells and during biofilm formation, respectively [20]–[22]. Both the transcriptome analysis of *A. pleuropneumoniae* exposed to bronchoalvolar fluid and our data suggested that the expression of genes involved in anaerobic energy generation and the synthesis of proteins involved in cell wall biogenesis were modulated in the early stages of infection [21]. We also cross-referenced our results to three other *in vivo* studies, identifying genes imperative for survival of *A. pleuropneumoniae*, in the host by STM and SCOTS, respectively [16]–[18], and against a study which used the method “high-throughput insertion tracking by deep sequencing” (HITS) for the identification of *H. influenzae* genes required for survival in a murine pulmonary model [25]. We identified 31 genes, which according to the various *in vivo* methods (STM, HITS, SCOTS), were important for bacterial survival in the host. Eight of these genes, e.g. the chaperone protein, *dnaK*, and the anaerobic dimethyl sulfoxide reductase, *dmsA*, were found in *H. influenzae*, which illustrates that some common strategies for survival in the host are shared among members of *Pasteurellaceae*.

From the previous studies shown in Table S5, however, no clear consensus emerges—indeed, the majority of loci identified across all seven studies were found in no more than two. The observed variation in outcome probably reflects the differences in methodology and bacterial strains used.

### Global gene regulation during infection

Possibly sRNAs, may be influencing the global pattern of bacterial gene regulation during anaerobic conditions in the host [69]. In that aspect, the global iron regulator Fur may be interesting, as there is an emerging picture of Fur as an important regulator, either directly or indirectly, of global RNA expression in bacteria. Present evidence suggests that Fur plays a global role in basic bacterial physiology and has a considerably wider impact on gene expression, at least in some bacterial species, than originally perceived [66], [70]. We also observed gene expression patterns *in vivo* which were consistent with active Fur-regulation, indicating that Fur could be governing functions influencing survival in the lung environment. Supporting this assumption is also the previous demonstration that a Fur mutant of *A. pleuropneumoniae* showed growth deficiencies *in vitro* and reduced virulence in an aerosol infection model [71].

### Secretion systems

In Gram-negative bacteria, specialized protein secretion systems are essential for transport of virulence factors, mainly toxins, adhesins and proteases, across the two membranes and into the extracellular environment [72], [73]. The Tat apparatus is well conserved among bacterial pathogens and appears to be involved in several virulence related traits such as iron uptake, anaerobic respiration, osmotic stress defense, copper homeostasis, motility and biofilm formation – all factors which are important for the pathogens ability to colonize and survive in the host [59], [74]. Our results, identifying 7 genes whose products are putatively exported by the Tat-pathway, indicated that this system also could be of importance for *A. pleuropneumoniae* pathogenesis.

### Metabolic adaptations to host environment

An interesting new observation in *A. pleuropneumoniae* expression during infection was the regulation of competence genes in early infection. This phenomenon has also been observed *in vivo* in other bacteria, such as *Listeria monocytogenes* and *Streptococcus pneumoniae* in animal models of infection [5], [75]. Extracellular DNA is highly abundant in natural environments, for example in lung mucus, and the ability to consume this extracellular DNA, as a source of nutrition or to increase genetic fitness, may convey enhanced survival for the bacteria [76].

After successful attachment, *A. pleuropneumoniae* requires nutrients provided by the host to grow and cause disease. Generally, the supply of essential nutrients is limited in the lower respiratory tract. *A. pleuropneumoniae* can overcome this problem by the induction of lysis of host cells by secreted exotoxins, resulting in the release of nutrients into the environment [14]. The combined effect of rapid bacterial proliferation, exotoxins and host immune factors probably results in extensive tissue destruction and the formation of fibrino-hemorrhagic lesions quite early in the infectious process. Such lesions are likely to represent an anaerobic environment [77]. The observed amount of differentially expressed genes involved in anaerobic metabolism, clearly indicated that the pathogen was experiencing anaerobic growth conditions quite early in the infection (Table 1).

Also worth noticing—and to our knowledge—not previously reported in *A. pleuropneumoniae* during *in vivo* infection, was the differential regulation of the Na+ pump, oxaloacetate decarboxylase (*oadAB*). Along with the simultaneous differential expression of the Na(+)/H(+) antiporter (*nhaB*), which play a major role in pH and Na(+) homeostasis, this was a strong indication that Na+ pumps were of importance during infection. In *H. influenza*, *nhaB* was among the genes required for growth and survival in a murine pulmonary model [25]; and up-regulation of *nqrB* and *nhaA* was also reported in *A. pleuropneumoniae* exposed to BALF [21]. Na+ gradient generation by decarboxylase-coupled ion transfer has only been identified in a limited number of (mostly) anaerobic bacteria, making it an exception rather than a rule in the microbial world [78]. Genes encoding primary Na+ pumps are found in the genomes of a number of phylogenetically diverse pathogenic bacteria. It is therefore quite possible that generation of a Na+ gradient is an important part of their membrane energetic, possibly constituting an alternative way of providing the bacteria with additional means of ATP synthesis, motility, and solute uptake which could improve its chances of colonization and survival in the host [78]. This study produced results which corroborate the findings of previous studies indicating that enzymes of anaerobic metabolism are essential for persistence of *A. pleuropneumoniae* in the host [79], [80].

Regulation of carbohydrate metabolism could be a challenging enterprise for *A. pleuropneumoniae* during the acute phase of the disease. Host cell lysis induced by secreted exotoxins may have released a surplus of carbohydrate sources from the host cells, as indicated by the observed expression pattern, where numerous genes involved in carbohydrate metabolism were differentially regulated (Table S3). A balanced carbon flux is extremely important for the viability of bacterial cells. DHAP is formed as an intermediate of glycolysis in both the activated pathways for anaerobic glycerol dissemination (*glp* and *dha*). Unfortunately for the bacteria, accumulation of triose phosphates has a growth limiting effect. This could be the explanation why *A. pleuropneumoniae* seemed to be engaged in the high risk strategy of activating the potentially suicidal methylglyoxal synthase (encoded by the *mgsA* gene), which produces MG from an excess surplus of DHAP [34]. The MG bypass could be an important mechanism for the bacteria in adaption to changes in environmental conditions, as it creates a small window of opportunity for adaption to nutrient imbalance caused by an excessive carbon intake [34]–[36]. MG appears to play a key role in the physiology of intracellular pathogens [36], exemplified by the identification of *mgsA* as important for *H. influenzae* survival in the murine host [25].

Down-regulation of iron uptake systems under anaerobic conditions is most likely caused by Fur-dependent repression [77]. As previously mentioned, we observed high expression of Fur during the first 48 h of infection. As Fur represses genes involved in iron uptake when levels of iron are high, this suggested that the porcine lung microenvironment offered sufficient amounts of iron for *A. pleuropneumonia*e to grow and multiply in this phase of infection [81]. Considering the hemorrhagic lesions from which the bacteria were isolated, it is quite possible that iron was not in short supply in these surroundings. Indeed we did not observe any differential or high expression of many of the well-characterized iron uptake systems, such as *tbpBA* and *hgbA* [82], [83]. Also supporting the theory of an iron-sufficient environment during acute infection was the substantial amount of iron-utilizing genes involved in anaerobic metabolism which were expressed *in vivo*.

### Coping with the host immune response

The initial interaction between *A. pleuropneumoniae* and the porcine host takes place on the epithelial lining of the respiratory tract, where the microbial intruder has to face the host's first line of defense. The innate immune response is active against a broad spectrum of microbial pathogens and operates before an antigenic (adaptive) immune response has developed [84]. Alveolar macrophages and polymorphonuclear leukocytes constitute the major defense mechanism of the distal airways against invading microorganisms [85]. In these phagocytes, antimicrobial peptides, hydrolytic enzymes or reactive oxygen intermediates are released [84]. Both alveolar macrophages and polymorphonuclear leukocytes are able to phagocytose *A. pleuropneumoniae*, but only polymorphonuclear leukocytes can effectively kill the pathogen [85]. *A. pleuropneumoniae* is able to survive for more than 90 min within alveolar macrophages, during which time liberation of RTX toxins from the bacteria can destroy these host immune cells. This capacity to survive within macrophages may be due to several factors such as capsule, LPS, stress proteins and ammonia [12].

Host-pathogen interaction was reflected in the active remodeling of the bacterial envelope through activation of genes responsible for cell wall components (Table 2). The cell wall and membrane may provide important protection against cell surface damaging factors of the host environment. The resistance of *A. pleuropneumoniae* to complement cytotoxicity, for example, can mainly be attributed to the capsular polysaccharide (*cps*) and/or LPS [12]. We didn't detect any significantly differential or high expression of the *cps* genes. In a previous investigation, the *cps* genes were found to be down-regulated in *A. pleuropneumoniae in vivo* [11]. In early infection, the expression of a thick capsule may be disadvantageous for the bacterium because of its inhibitory effect on adherence [11], [86]. This phenomenon has also been observed in other lung pathogens such as *S. pneumonia*e, where expression of a thinner capsule promotes binding to host tissue during initial stages of colonization [87].

In *A. pleuropneumoniae*, host contact may have induced significant regulation or high expression of genes coding for key enzymes in LPS biosynthesis (*kdsB*), the peptidoglycan biosynthetic pathway (*murDI*), lipoprotein E synthesis (*ompP4*), enterobacterial common antigen (ECA) (*wecBCDE*) and ompP5 (*ompA*). In *H. influenzae*, lipoprotein E is essential for hemin uptake and the utilization of Nicotinamide adenine dinucleotide [45], [88], [89]. As *A. pleuropneumoniae* biotype-1 is also dependent on exogenous sources of Nicotinamide adenine dinucleotide for growth [90], lipoprotein E could also be relevant for the pathogenesis of this bacterium.

In non-typable *H. influenzae* (NTHI), OmpP5 binds specifically to a variety of receptors on the host cell membrane, including respiratory mucin and bronchial epithelial cells [91]. It is interesting to note, that in NTHI, OmpP5-derived peptides provide significant protection against homologous and heterologous NTHI challenge in chinchilla and rat models of otitis media [46]. Previously, *APL_1421* has been reported to be expressed in necrotic porcine lung tissue by SCOTS [18], while an *APL_1852* homolog was among the genes required by *H. influenzae* for survival in the murine lung [25]. Finally, a new study identified both OmpP5 proteins as immunoreactive in sera from swine naturally infected with *A. pleuropneumoniae* serotype 1 and in hyperimmune sera raised in an immunized rabbit [48]. This corresponds well with the findings in the current study, where both *APL_1421* and *APL_1852* were found constitutively highly expressed.

From the *in vivo* induction of a number of stress genes, we were able to make inferences concerning the stress factors *A. pleuropneumoniae* is facing in the host and the genes that were mobilized in order to survive in this harsh environment. Our results indicated that the pathogen encountered—and were able to cope with—host immune factors, e.g. reactive oxygen intermediates, produced by the alveolar macrophages as well as toxic concentrations of copper. Also, we observed significant differential expression of the genes *ureADEG*, encoding urease subunits which may be involved in resistance to macrophage damage and bacterial chronic infection [52], [53].

Sialic acid may function as an anti-recognition molecule, modifying the bacterial cell surface to mimic the host cell surface and subvert or inhibit host innate immunity [56], [92]. This function has been implicated as a virulence factor in several bacterial species [56]. For example, LPS sialylation is a feature of several pathogenic members of the *Pasteurellaceae*, including *H. ducreyi*, *H. influenzae*, *Histophilus somni* and *P. multocida* [93]. The significant regulation of acylneuraminate cytidylyltransferase (*neuA*) leaves open the possibility that *A. pleuropneumoniae* could be using a mechanism of cell surface sialyation, called precursor scavenging. This method is also applied by *H. influenzae*, which, like *A. pleuropneumoniae*, lacks *neuBC* but has orthologues of *neuA* [92], [94]. In *H. influenzae*, sialyated LPS glycoforms play a key role in pathogenicity of nontypeable variants which scavenge the essential precursors from the host during the infection shown in a chinchilla model of otitis media [92]. For *P. multocida*, sialylation appears to be necessary for systemic pasteurellosis, presumably by protecting the sialylated bacteria from innate host defense mechanisms [95]. Further investigations are necessary to clarify the potential influence of sialic acid cell surface modifications upon the virulence of *A. pleuropneumoniae*.

### Concluding remarks

The outcome of a bacterial infection is determined by the complex interactions of multiple host and microbial factors. The physiochemical elements of this relationship are very difficult to reproduce under *in vitro* conditions. By monitoring the bacterial *in vivo* genomic expression during the first critical phase of infection in its natural host, we were able to derive new detailed information regarding host-pathogen interactions. Data presented here illustrated how *A. pleuropneumoniae* was able to adapt its metabolism to derive carbon and energy from an anaerobic environment and how the microbe was employing a broad range of strategies to evade and counteract the effects of the host immune response. Understanding the metabolic basis of bacterial pathogenesis may provide a rational basis for the development of new therapeutical strategies. Many new targets for future research have been uncovered and future phenotypic analysis will show if some of the potential virulence genes identified here may serve as new targets in drug and vaccine development.

## Materials and Methods

### Ethics statement

All animal procedures were approved by the Danish Animal Experiments Inspectorate under the Ministry of Justice (permit number: 2006/561-1106) and the animal experiments were conducted in strict accordance with their guidelines.

### Bacterial strains and growth conditions

*A. pleuropneumoniae* serotype 2 (4226) and serotype 6 (7712640), both Danish field strains isolated from pigs with acute pleuropneumonia, were used for the infection studies. *A. pleuropneumoniae* was grown on PPLO agar plates (Difco) at 37°C overnight and subsequently resuspended in 0.9% NaCl and adjusted to a density corresponding to McFarland standard 1. This suspension was mixed 1∶1 with Brain heart infusion broth (Difco) added 5% NAD and used for infection.

### Infection studies

For *A. pleuropneumoniae* serotype 2 and 6, respectively, 24 8–10-week-old Danish specific pathogen free (SPF) piglets were infected via the intranasal route. Due to practical reasons, the infection experiments for each of the serotypes were performed on separate days. The animals received 2 ml of a bacterial suspension containing a total dose of 2×10^8^ CFU or 1×10^8^ CFU of serotype 2 or 6, respectively. Six animals were sacrificed 6 h, 12 h, 24 h and 48 h post infection, respectively. The animals were sedated with Zoletil ® (Virbac, Carros, France) and Narcoxyl ® Intervet MSD Animal Health (Ballerup, Denmark) and euthanized with pentobarbital (Veterinærapoteket, University of Copenhagen, Denmark). Of the 48 pigs one was euthanized due to unrelated neck infection and another two succumbed to serotype 2 infections before sampling. In total 28 animals displayed visual lung lesions (Table S1). Immediately post mortem, infected lung tissue was isolated, cut into pieces smaller than 0.5×0.5 cm and preserved in RNA*later* stabilization reagent (Ambion, Cambridgeshire, United Kingdom) at −20°C. Samples from the lungs were cultivated on PPLO agar (SSI, Copenhagen, Denmark) and Columbia agar plates (Oxoid, Greve, Denmark) with 5% calf blood added at 37°C in atmospheric air over night to re-isolate the inoculation strain and other bacteria present. Bacterial identification was done according to the standard procedures of the laboratory.

### RNA isolation and reverse transcription

Total RNA was isolated from 100 to 300 mg lung tissue with visual lesions (3 samples from each animal). Prior to RNA extraction using the RNeasy Lipid kit (QIAGEN, Hilden, Germany), the tissue was finely chopped by scalpel, transferred to 5 ml Phenol:Guanidinthiocyanat lysis buffer (provided in the Qiagen kit) in which it was divided further by a Tissue-Tearor, 985370-XL (BioSpec Products, Bartlesville, OK) for 2 min. The remaining steps of the RNA extraction was performed according to the protocol provided in the kit (Qiagen). Genomic DNA was eliminated by RNase-free DNase I treatment during the isolation procedure. After RNA extraction, the material was further treated by TURBO™ DNase, according to the protocol provided by the manufacturer (Ambion). At this point no trace of bacterial or host DNA could be detected in the qPCR analysis. The RNA concentration and quality were measured by NanoDrop (Thermo Scientific, Wilmington, DE, USA) and Agilent 2100 Bioanalyzer (Agilent Technologies, Santa Clara, CA), respectively. Quality requirements were: A260/A280≥1.8 and RIN>5. Samples not meeting this standard were discarded and new extractions performed. Most of the RIN scores were between 6 and 7, which indicated some degree of RNA degradation. However, as the integrity number was based on a mixture of pro-, and eukaryotic RNA, with the latter comprising main part of the measured RNA, we assumed that this was primarily due to increased enzymatic processes in the infected eukaryotic tissue which would most likely not affect the integrity of bacterial RNA protected by the bacterial cell wall.

For each sample, 30 µg of total RNA was enriched for bacterial RNA applying the MicrobEnrich Kit according to the supplied protocol (Ambion). Subsequently, one µg of the enriched RNA was amplified using a MessageAmp II-Bacteria kit (Ambion) according to the manufacturer's instructions.

### Preparation of labeled double-stranded DNA

Ten micrograms of total RNA from each sample was reverse transcribed using SuperScript II (Invitrogen, Carlsbad, CA) and Random Hexamer Primers (Invitrogen) according to the NimbleGen Arrays User's Guide (Gene Expression Analysis v3.2). The generated cDNA was incubated with 1 µl of 4 mg/ml RNase A solution (Promega Corporation, Madison, WI) at 37°C for 10 min, and then phenol-chloroform extracted. Samples were centrifuged in Phase Lock Gel Tubes (5 Prime, Hamburg, Germany) at 12,000× g for 5 minutes and precipitated with 80% ethanol. Pellets were air dried in a SpeedVac and rehydrated in 20 µl of ultrapure water (Ambion). Finally the samples were measured by NanoDrop to ensure that the cDNA met the following quality requirements: A260/A280≥1.8 and A260/A230≥1.8. NimbleGen One-Color DNA Labeling kit (NimbleGen Systems, Madison, WI) was used for Cy3 labeling of cDNA samples according to the NimbleGen Arrays User's Guide. Briefly, 1 µg double-stranded cDNA was incubated for 10 min at 98°C with Cy3-random Nonamers and then quick-chilled in an ice-water bath for 10 min. The addition of 100 mM of deoxynucleoside triphosphates and 100 U of Klenow fragment (New England Biolabs, Ipswich, MA) was followed by incubation at 37°C for 2 h. The reaction was stopped by adding 0.1 volumes of 0.5 M EDTA, and the labeled cDNA was precipitated with isopropanol.

### DNA microarrays

The arrays used in this project were based on the NimbleGen 12-plex platform, officially released in a news statement on Nov. 19, 2008. The custom probe set for the arrays was build around a set of 7 core genomes representing all publically available *A. pleuropneumoniae* and *Actinobacillus succinogenes* genomes in GenBank and RefSeq, which included draft genome sequences of *A. pleuropneumoniae* serotypes 2 and 6 [PMID: 18073190]. The array included 130,194 active probes excluding NimbleGen control probes. Each gene was covered by an average of 26.7 probes of an average size of 48 bp. The detailed construction of the array has been previously described [96] and the design is publicly available at NimbleGen (091013_DTU_Actino_xRNA).

### Hybridization and analysis of arrays

A hybridization kit (NimbleGen Systems) was used for the hybridization step. Cy3-labeled samples were resuspended in the recommended amount of hybridization buffer and denatured at 95°C for 5 min. Slides were placed in HX12 NimbleGen Mixer and 6 µl of sample loaded though the fill port. Hybridization was performed for 20 h at 42°C (NimbleGen Hybridization System 16). The arrays were washed using a wash buffer kit (NimbleGen Systems), dried in a microarray dryer (NimbleGen Systems), and scanned at a 5 µm resolution using the NimbleGens MS 200 scanner (NimbleGen Systems).

### Quantitative real-time PCR (qPCR)

Gene quantification was performed with a Rotor-Gene 6000 (Corbett Research, Sydney, Australia). The primers were designed using Primer3 (v. 0.4.0) [97]. The sequences of the primers are listed in Table S6. Each PCR was performed in a 25 µl reaction mixture containing 12.5 µl QuantiTect SYBR Green PCR master mix (Qiagen, Hilden, Germany), a primer concentration of 0.3 µM and 7 ng of cDNA. Three biological replicas were included for each sample. The thermal cycling conditions were as follows: 15 min at 95°C, followed by 40 cycles of 30 s at 94°C, 20 s at 55°C, and 20 s at 72°C. Data collection was performed during each extension phase. Positive controls (DNA) and negative controls (distilled water) were included in each run. Control for DNA contamination was performed before linear amplification of the mRNA. Melting curve analysis was performed, which for all primer sets resulted in single product-specific melting curves.

In the qPCR analysis of amplified versus non-amplified *A. pleuropneumoniae* mRNA the target concentration for each amplicon was determined from an optimized standard curve (Table S7). The concentration 3600 pg/µl was used as inter-plate calibrator. The geometric means of the previously validated genes *glyA* and *pykA* were applied as reference genes for normalization [98].

For validation of the microarray data relative quantification was applied. The Excel-based relative expression software tool, REST 2009 (V2.0.13), was applied for group wise comparison and statistical analysis of the qPCR data (http://rest.gene-quantification.info/) [99]. The relative expression ratios were calculated by a mathematical model, which included an efficiency correction for real-time PCR efficiency of the individual transcripts [100]:
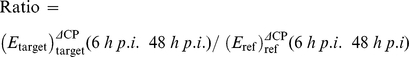



The relative expression ratio of a target gene was computed based on its real-time PCR efficiencies (*E*) and the crossing point difference (ΔCP) for an unknown sample versus a control (in this case we compared 6 hours p. i. versus 48 hours p.i.). For each gene, cDNA dilution curves were generated and used to calculate the individual real-time PCR efficiencies (*E* = 10^[−1/slope]^). The geometric mean of two internal reference genes was used to correct the raw values for the genes of interest (Table S7). From the genes that displayed the least variations in expression between the 75 microarrays, two new references were chosen for normalization. These were the carbon storage regulator (*csrA*), belonging to the functional group “signal transduction mechanisms” and phosphomannomutase (*manB*) from the functional group “carbohydrate transport and metabolism”.

### Microarray analysis

The data discussed in this publication have been deposited in NCBI's Gene Expression Omnibus [101] and are accessible through GEO Series accession number GSE33999. (http://www.ncbi.nlm.nih.gov/geo/query/acc.cgi?acc=GSE33999). Data analysis of the microarrays was performed in “RGui” version 2.9.2 (2009-08-24) (http://cran.r-project.org/bin/windows/base/), using the package “Oligo”. The Robust Multichip Average function was applied for normalization and index calculation of the microarray data [102]. By this method, the expression measure is given in log_2_ base. For each time point, the mean log_2_ expression values of the included pigs (three samples from each pig) are given in Table 1 and Table 2. A two-way analysis of variance (ANOVA) was used on the entire dataset to test the effect of serotype (F1: *A. pleuropneumoniae* serotype 2 versus serotype 6) and time (F2: variations between time points 6 h, 12 h, 24 h and 48 h p.i.) (Table S3).

Figure S1 shows a density plot of the 75 microarrays. Probe targets where no hybridization signal was detected (signal below 9 for all samples or one of the serotypes) were omitted from the analysis.

Functional classification of the ORFs identified to be either differentially expressed or constitutively highly expressed during the first 48 hours of infection was performed with the Entrez Protein Clusters database (http://www.ncbi.nlm.nih.gov/proteinclusters) [24].

## Supporting Information

Figure S1 **Density plot of the expression profiles from all 75 arrays used in this study.** Each colored line reveals the distribution of signal for one specific array. The tall spike to the left clearly indicates genes either not present in the organism or not expressed at all. The softer hill-like spike to the right represents those genes experiencing at least a certain level of expression. Created using the density() function in R, which uses Fourier transformations and Gaussian kernel estimates to derive the functions underlying the observed data.
(PDF)

Table S1 Overview of pig samples included in the study.
(PDF)

Table S2 Quantitative RT-PCR analysis of amplified versus non-amplified samples.
(PDF)

Table S3 *A. pleuropneumoniae* genes displaying significant differential expression during the acute phase of infection.
(PDF)

Table S4 The most highly and constitutively expressed genes of *A. pleuropneumoniae* during the first 48 h post experimental challenge.
(PDF)

Table S5 Genes found to be differentially expressed in the present and other expression studies of *A. pleuropneumoniae* or *H. influenzae*.
(PDF)

Table S6 List of primers used for quantitative real-time PCR.
(PDF)

Table S7 R^2^ values and Efficiency of standard curves used in qPCR analyses.
(PDF)
