# Supplementary figures and images for: Transcriptional Portrait of Actinobacillus pleuropneumoniae during Acute Disease - Potential Strategies for Survival and Persistence in the Host
Source: PLoS One. 2012 Apr 17;7(4):e35549. doi: 10.1371/journal.pone.0035549 (PMC3328466; doi:10.1371/journal.pone.0035549)

Density of 75 arrays

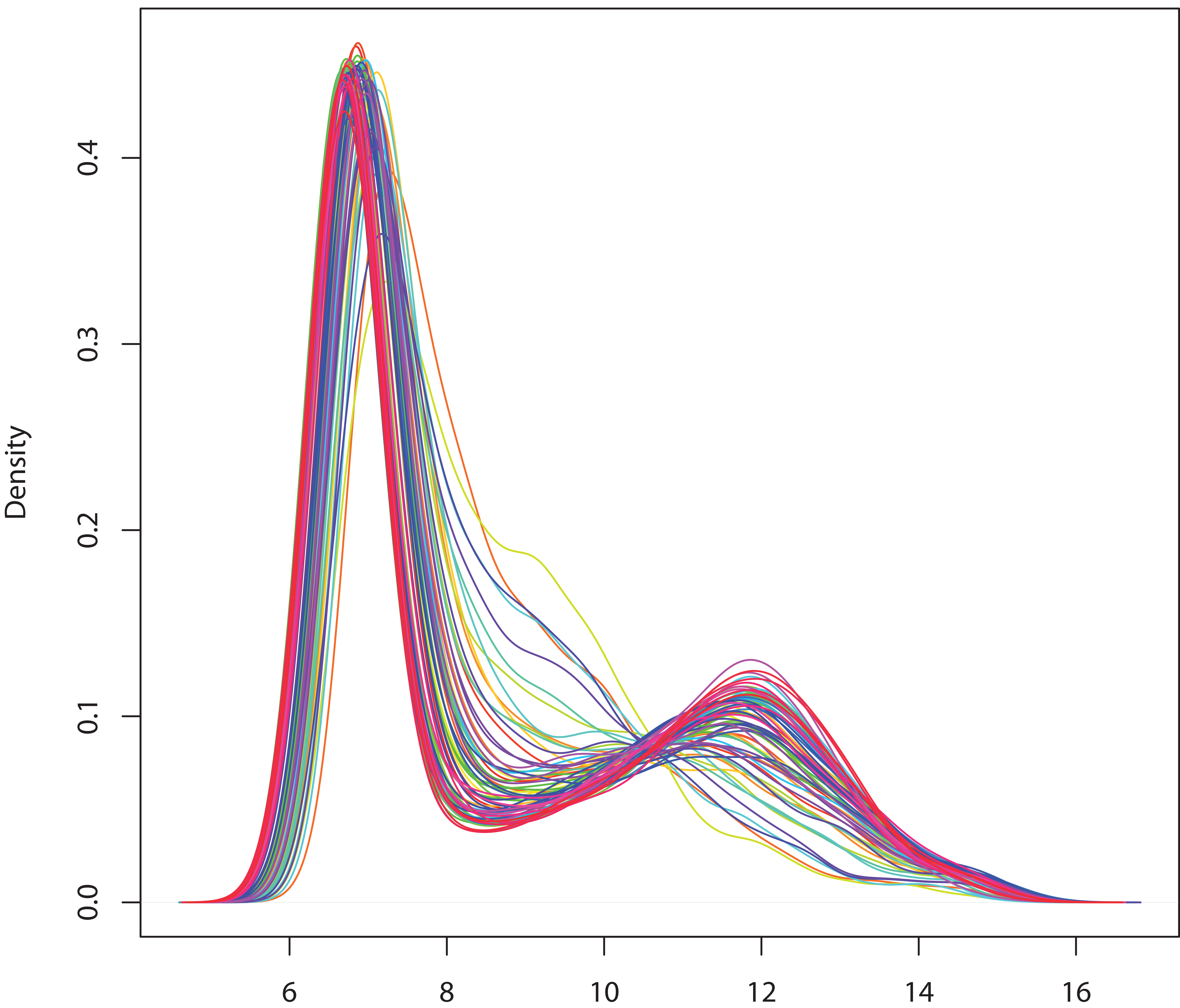

N = 4876 Bandwidth = 0.3913

Supplement: Figure S1 — Density plot of the expression profiles from all 75 arrays used in this study. Each colored line reveals the distribution of signal for one specific array. The tall spike to the left clearly indicates genes either not present in the organism or not expressed at all. The softer hill-like spike to the right represents those genes experiencing at least a certain level of expression. Created using the density() function in R, which uses Fourier transformations and Gaussian kernel estimates to derive the functions underlying the observed data. (PDF) [file pone.0035549.s001.pdf]
