# Supplementary material for: Transcriptional Portrait of Actinobacillus pleuropneumoniae during Acute Disease - Potential Strategies for Survival and Persistence in the Host
Source: PLoS One. 2012 Apr 17;7(4):e35549. doi: 10.1371/journal.pone.0035549 (PMC3328466; doi:10.1371/journal.pone.0035549)
Supplement: Table S1 — Overview of pig samples included in the study. (PDF) [file pone.0035549.s002.pdf]

**Table S1.** Overview of pig samples included in the study

| Pig no. | Time post infection | Serotype | Bacteria cultivated from lung |
|---|---|---|---|
| 33 | 6 | Ap6 | *A. pleuropneumoniae* serotype 6 in mixed culture with contaminating flora |
| 36 | 6 | Ap6 | *A. pleuropneumoniae* serotype 6 in mixed culture with *S. suis* serotype 7 |
| 38 | 12 | Ap6 | *A. pleuropneumoniae* serotype 6 in pure culture |
| 39* | 12 | Ap6 | *A. pleuropneumoniae* serotype 6 in mixed culture with contaminating flora |
| 40 | 12 | Ap6 | *A. pleuropneumoniae* serotype 6 in mixed culture with *S. suis* serotype 7 and *P. multocida* |
| 43* | 24 | Ap6 | *A. pleuropneumoniae* serotype 6 in pure culture |
| 46 | 24 | Ap6 | *A. pleuropneumoniae* serotype 6 in pure culture |
| 48 | 24 | Ap6 | *A. pleuropneumoniae* serotype 6 in pure culture |
| 50 | 48 | Ap6 | *A. pleuropneumoniae* serotype 6 with non-haemolytic *E. coli* and *S. suis* serotype 8. |
| 51 | 48 | Ap6 | *P. multocida* and *S. suis* serotype 7 in mixed culture, PCR proved presence of *A. pleuropneumoniae* serotype 6 |
| 54 | 48 | Ap6 | *A. pleuropneumoniae* serotype 6, in mixed culture with *P. multocida* and *S. suis* serotype 8 |
| 55 | 6 | Ap2 | *A. pleuropneumoniae* serotype 2, in mixed culture with *P. multocida* |
| 56 | 6 | Ap2 | *A. pleuropneumoniae* serotype 2, in mixed culture with *P. multocida* |
| 57 | 6 | Ap2 | *A. pleuropneumoniae* serotype 2, in mixed culture with *P. multocida* |
| 58 | 6 | Ap2 | *A. pleuropneumoniae* serotype 2, in mixed culture with *P. multocida* |
| 59 | 6 | Ap2 | *A. pleuropneumoniae* serotype 2, in mixed culture with *P. multocida* |
| 60 | 12 | Ap2 | *A. pleuropneumoniae* serotype 2, in mixed culture with *P. multocida* |
| 62 | 12 | Ap2 | *A. pleuropneumoniae* serotype 2, in mixed culture with *P. multocida* |
| 66 | 12 | Ap2 | *A. pleuropneumoniae* serotype 2, in mixed culture with *P. multocida* |
| 67 | 24 | Ap2 | *A. pleuropneumoniae* serotype 2 in mixed culture with non-hemolytic *E. coli* and *P. multocida* |
| 69 | 24 | Ap2 | *A. pleuropneumoniae* serotype 2, in mixed culture with *P. multocida* |
| 70 | 24 | Ap2 | *A. pleuropneumoniae* serotype 2, in mixed culture with *P. multocida* |
| 71 | 24 | Ap2 | *A. pleuropneumoniae* serotype 2 in mixed culture with *P. multocida* and *S. suis* |
| 72* | 24 | Ap2 | *A. pleuropneumoniae* serotype 2 in pure culture |
| 73 | 48 | Ap2 | *A. pleuropneumoniae* serotype 2 in mixed culture with *P. multocida* |
| 75 | 48 | Ap2 | *A. pleuropneumoniae* serotype 2 in mixed culture with non-hemolytic *E. coli* , *P. multocida* |
| 76 | 48 | Ap2 | *A. pleuropneumoniae* serotype 2 in mixed culture with *P. multocida* and *S. suis* |
| 78 | 48 | Ap2 | *A. pleuropneumoniae* serotype 2 in mixed culture with *P. multocida* |

*Samples excluded from the array analysis due to lack of hybridization signal.
