# Supplementary material for: Transcriptional Portrait of Actinobacillus pleuropneumoniae during Acute Disease - Potential Strategies for Survival and Persistence in the Host
Source: PLoS One. 2012 Apr 17;7(4):e35549. doi: 10.1371/journal.pone.0035549 (PMC3328466; doi:10.1371/journal.pone.0035549)
Supplement: Table S2 — Quantitative RT-PCR analysis of amplified versus non-amplified samples. (PDF) [file pone.0035549.s003.pdf]

Table S2. Quantitative RT-PCR analysis of amplified versus non-amplified samples

	**Sample no. 33**		**Sample no. 55**	
	Non-amplified . $\log_2$ mean expression (n=3)	**Amplified mRNA. $\log_2$ mean expression (n=3)**	**Non-amplified . $\log_2$ mean expression (n=3)**	**Amplified ($\log_2$) Mean expression ($\log_2$)**
glyA	0.87	0.87	0.68	0.74
pykA	1.13	1.13	1.02	1.02
tpiA	1.20	1.09	0.98	0.98
luxS	0.68	0.87	0.53	0.71
cirA	0.70	0.85	0.50	0.74
hybB	0.91	0.85	0.56	0.60
fdxG	0.92	1.02	0.81	0.87
tonB1	0.78	0.73	0.58	0.60
hlyX	0.85	0.87	0.89	0.78
hyaA	0.44	0.76	0.06	0.52
tbpA1	0.92	0.87	0.82	0.76
tonB2	0.26	0.78	0.12	0.62
lldD	0.32	0.73	0.21	0.63
