# Supplementary material for: Transcriptional Portrait of Actinobacillus pleuropneumoniae during Acute Disease - Potential Strategies for Survival and Persistence in the Host
Source: PLoS One. 2012 Apr 17;7(4):e35549. doi: 10.1371/journal.pone.0035549 (PMC3328466; doi:10.1371/journal.pone.0035549)
Supplement: Table S3 — A. pleuropneumoniae genes displaying significant differential expression during the acute phase of infection. (PDF) [file pone.0035549.s004.pdf]

**Table S3.** *A. pleuropneumoniae* genes displaying significant differential expression during the acute phase of infection

| Gene designation | Locus no.# | Annotation | 6 h ($Log_2$)* | 12 h ($Log_2$)* | 24 h ($Log_2$)* | 48 h ($Log_2$)* | P-value |
|---|---|---|---|---|---|---|---|
| *Energy production and conversion* | | | | | | | |
| hyaA | APL_1331 | Hydrogenase 2 small subunit | 10.03 | 9.15 | 8.3 | 7.82 | 2.55E-22 |
| hybA | APL_1332 | Hydrogenase 2 protein HybA | 11 | 10.26 | 9.26 | 8.81 | 1.75E-21 |
| lpdA | APL_0771 | Dihydrolipoyl dehydrogenase | 11.68 | 11.03 | 10.23 | 9.61 | 3.88E-19 |
| hyaD | APL_1335 | Hydrogenase 2 maturation protease | 12.46 | 11.95 | 11.18 | 10.8 | 3.99E-18 |
| torZ | APL_0688 | Trimethylamine-N-oxide reductase precursor | 12.73 | 12.13 | 11.31 | 10.54 | 1.21E-16 |
| glpQ | APL_0378 | Glycerophosphoryl diester phosphodiesterase | 10.83 | 10.04 | 9.21 | 8.43 | 4.71E-16 |
| hyaB | APL_1334 | Hydrogenase-2 large chain | 12.24 | 11.69 | 10.4 | 9.94 | 2.46E-15 |
| torY | APL_0689 | Cytochrome c-type protein | 12.66 | 12.13 | 11.23 | 10.44 | 2.82E-15 |
| acyP | APL_0143 | Acylphosphatase | 13.4 | 12.99 | 12.35 | 12.15 | 1.24E-14 |
| dcuC | APL_0870 | Putative C4-dicarboxylate transporter | 11.21 | 11.04 | 10.12 | 9.46 | 6.39E-14 |
| hybB | APL_1333 | Putative Ni/Fe-hydrogenase 2 b-type cytochrome subunit | 12.03 | 11.64 | 10.39 | 9.72 | 6.76E-14 |
| glpA | APL_0379 | Sn-glycerol-3-phosphate dehydrogenase subunit A | 11.18 | 10.93 | 10.48 | 9.94 | 1.96E-13 |
| yfhL | APL_0058 | Ferredoxin-like protein | 10.98 | 10.21 | 9.09 | 8.62 | 8.24E-11 |
| nfnB | APL_0607 | Putative NAD(P)H nitroreductase | 11.47 | 10.74 | 10.15 | 9.55 | 1.04E-10 |
| glpK | APL_0375 | Glycerol kinase | 12.36 | 12.25 | 11.41 | 11.13 | 1.38E-10 |
| pckA | APL_0800 | Phosphoenolpyruvate carboxykinase [ATP] | 13.4 | 13.26 | 12.51 | 12.34 | 4.51E-10 |
| APL_1591 | APL_1591 | Flavodoxin/protoporphyrinogen oxidase | 11.92 | 11.5 | 10.32 | 10.34 | 8.46E-10 |
| maeB | APL_0486 | NADP-dependent malic enzyme (NADP-ME) | 10.09 | 9.94 | 9.42 | 9.34 | 8.87E-10 |
| nrfC | APL_0102 | Nitrate reductase | 12.41 | 12.47 | 11.19 | 10.92 | 1.20E-09 |
| dmsA | APL_1674 | Anaerobic dimethyl sulfoxide reductase chain A precursor | 13.17 | 13.32 | 12.3 | 11.7 | 1.34E-09 |
| oadB | APL_1377 | Oxaloacetate decarboxylase beta chain | 11.45 | 11.42 | 10.54 | 10.01 | 1.46E-09 |
| oadA | APL_1376 | Oxaloacetate decarboxylase alpha chain | 11.25 | 11.1 | 10.17 | 9.78 | 1.59E-09 |
| *Cell cycle control, mitosis and meiosis* | | | | | | | |
| crcB | APL_1533 | Protein CrcB homolog | 10.77 | 10.37 | 9.99 | 9.65 | 1.85E-10 |
| fstE | APL_1345 | Cell division ATP-binding protein | 11.11 | 10.86 | 10.28 | 9.83 | 4.92E-10 |
| ftsN | APL_1870 | Possible cell division protein | 11.87 | 11.36 | 10.67 | 10.54 | 5.36E-10 |
| *Amino acid transport and metabolism* | | | | | | | |
| nanA | APL_1754 | N-acetylneuraminate lyase | 11.85 | 11.57 | 9.73 | 9.32 | 1.37E-16 |
| ansB | APL_0135 | L-asparaginase II | 12.38 | 11.94 | 11.37 | 10.87 | 1.49E-15 |
| APL_0848 | APL_0848 | Putative ABC transporter periplasmic binding protein | 11.58 | 11.5 | 9.41 | 8.98 | 2.12E-15 |
| leuC | APL_0139 | 3-isopropylmalate dehydratase large subunit 2 | 9.32 | 8.66 | 8.42 | 8.26 | 5.83E-15 |
| lysA | APL_1525 | Diaminopimelate decarboxylase | 10.22 | 9.81 | 9.32 | 8.93 | 3.97E-14 |
| proB | APL_0884 | Glutamate 5-kinase | 10 | 9.51 | 8.99 | 8.43 | 1.04E-13 |
| APL_0849 | APL_0849 | Inner membrane ABC transporter permease | 9.38 | 9.22 | 7.59 | 7.43 | 1.92E-13 |
| ureA | APL_1618 | Urease subunit gamma | 10.33 | 9.64 | 9.45 | 9.16 | 3.19E-13 |
| pepA | APL_1101 | Probable cytosol aminopeptidase | 11.73 | 11.45 | 10.67 | 10.19 | 8.19E-13 |
| thrC | APL_1499 | Threonine synthase | 11.34 | 10.88 | 10.24 | 9.92 | 8.54E-13 |
| neuA | APL_1751 | Acylneuraminate cytidylyltransferase | 10.31 | 9.97 | 8.41 | 8.03 | 2.54E-12 |
| ilvE | APL_0072 | Branched-chain amino acid aminotransferase | 12.32 | 12.07 | 11.47 | 11.12 | 3.22E-12 |
| artM2 | APL_1604 | ABC-type amino acid transport system | 9.35 | 8.8 | 8.54 | 7.98 | 4.07E-12 |
| pabB | APL_1145 | Para-aminobenzoate synthase component I | 9.18 | 8.93 | 8.75 | 8.57 | 7.11E-12 |

| | | | | | | | |
|---|---|---|---|---|---|---|---|
| APJL_1980 | APJL_1980 | Carboxypeptidase G2 | 11.5 | 11.21 | 10.25 | 9.43 | 1.11E-11 |
| lysE | APL_1046 | Lysine exporter protein | 9.06 | 8.27 | 7.95 | 7.51 | 1.96E-11 |
| dapF | APL_1531 | Diaminopimelate epimerase | 10.93 | 10.38 | 9.82 | 9.28 | 1.28E-10 |
| proA | APL_1951 | Gamma-glutamyl phosphate reductase | 10.84 | 10.68 | 10.38 | 10.02 | 1.99E-10 |
| APL_0701 | APL_0701 | O-acetylhomoserine (Thiol)-lyase | 11.38 | 11.04 | 10.7 | 10.45 | 3.57E-10 |
| glnB | APL_1518 | Nitrogen regulatory protein P-II | 10.72 | 10.25 | 9.51 | 9.01 | 3.92E-10 |
| dut | APL_1968 | Deoxyuridine 5'-triphosphate nucleotidohydrolase | 10.26 | 9.71 | 9.17 | 8.36 | 7.81E-10 |
| *Nucleotide transport and metabolism* | | | | | | | |
| cpdB | APL_0646 | 2',3'-cyclic-nucleotide 2'-phosphodiesterase | 10.54 | 10.18 | 9.75 | 9.51 | 1.23E-14 |
| cdd* | APL_1343 | Cytidine deaminase | 10.53 | 9.95 | 9.46 | 9.12 | 1.32E-10 |
| prsA | APL_0775 | Ribose-phosphate pyrophosphokinase | 12.71 | 12.59 | 12.12 | 11.98 | 2.04E-10 |
| mutT | APL_0241 | Mutator mutT protein | 10.78 | 10.23 | 9.93 | 9.53 | 3.91E-10 |
| *Carbohydrate transport and metabolism* | | | | | | | |
| rbsB | APL_1672 | D-ribose-binding periplasmic protein | 12.2 | 11.53 | 10.79 | 10.39 | 4.26E-19 |
| dhaK | APL_0083 | PTS-dependent dihydroxyacetone kinase | 11.37 | 11.1 | 9.94 | 9.08 | 1.29E-17 |
| nagB | APL_1755 | Glucosamine-6-phosphate deaminase | 11.55 | 11 | 9.12 | 8.78 | 4.22E-17 |
| galK | APL_0995 | Galactokinase | 10.04 | 9.53 | 9.23 | 8.93 | 6.87E-16 |
| lacZ | APL_0997 | Beta-galactosidase | 9.85 | 9.11 | 8.64 | 8.19 | 2.32E-15 |
| APL_0707 | APL_0707 | Putative uncharacterized protein | 12.03 | 11.63 | 10.53 | 10.4 | 2.46E-15 |
| glpT | APL_0377 | glycerol-3-phosphate transporter | 13.07 | 12.99 | 12.02 | 11.54 | 5.36E-15 |
| xylF | APL_1909 | D-xylose transporter subunit XylF | 9.96 | 8.92 | 8.52 | 8.23 | 5.59E-15 |
| xylA | APL_1908 | Xylose isomerase | 9.22 | 8.54 | 8.14 | 8.02 | 8.21E-15 |
| mgsA | APL_1498 | Methylglyoxal synthase | 11 | 10.07 | 9.69 | 9.23 | 8.34E-15 |
| mglA | APL_1419 | Galactoside transport ATP-binding protein | 10.65 | 10.03 | 9.27 | 8.96 | 1.06E-14 |
| nanE | APL_1752 | Putative N-acetylmannosamine-6-phosphate 2-epimerase | 12.16 | 11.86 | 10.31 | 9.86 | 1.27E-14 |
| glpF | APL_0374 | Glycerol uptake facilitator protein | 11.16 | 10.96 | 10.42 | 9.73 | 2.33E-14 |
| rbsK | APL_1673 | Ribokinase | 11.76 | 11.41 | 10.88 | 10.65 | 3.23E-14 |
| dhaL | APL_0082 | PTS-dependent dihydroxyacetone kinase | 12.55 | 12.3 | 10.86 | 10.42 | 4.31E-14 |
| ulaC | APL_1699 | Ascorbate-specific phosphotransferase enzyme | 9.83 | 9.02 | 8.88 | 8.74 | 9.09E-14 |
| malG | APL_1239 | Maltose transport system permease protein | 9.88 | 9.53 | 8.83 | 8.04 | 3.59E-13 |
| xylG | APL_1910 | D-xylose transport ATP-binding protein | 9.32 | 8.57 | 8.06 | 7.82 | 1.47E-12 |
| nagA | APL_1756 | N-acetylglucosamine-6-phosphate deacetylase | 12.46 | 12.07 | 11 | 10.76 | 7.29E-12 |
| malK | APL_1236 | Maltose/maltodextrin import ATP-binding protein | 10.8 | 10.61 | 9.42 | 8.72 | 1.08E-11 |
| zwf | APL_1311 | Glucose-6-phosphate 1-dehydrogenase | 10.52 | 10.36 | 10.03 | 9.68 | 2.68E-11 |
| gntK | APL_1666 | Thermosensitive gluconokinase | 10.15 | 9.15 | 8.77 | 8.38 | 4.06E-11 |
| rpe | APL_1820 | Ribulose-phosphate 3-epimerase | 9.31 | 8.92 | 8.29 | 7.96 | 6.00E-11 |
| ulaD | APL_1698 | Probable 3-keto-L-gulonate-6-phosphate decarboxylase | 11.92 | 10.91 | 10.38 | 10.27 | 1.05E-10 |
| mglB | APL_1420 | D-galactose-binding periplasmic protein | 12.88 | 12.42 | 11.61 | 11.48 | 1.63E-10 |
| xylH | APL_1911 | ABC-type xylose transport system, permease component | 10.19 | 9.69 | 9.43 | 9.14 | 1.92E-10 |
| mtlA | APL_1630 | PTS system mannitol-specific EIICBA component | 12.09 | 11.65 | 11.11 | 11.01 | 2.27E-10 |
| ycaD | APP7_0851 | Uncharacterized MFS-type transporter | 11.38 | 12.22 | 12.45 | 12.77 | 4.01E-10 |
| pgm | APL_0591 | Phosphoglucomutase/phosphomannomutase | 12.43 | 12.25 | 11.69 | 11.41 | 4.34E-10 |
| glxK | APL_0142 | Glycerat kinase | 11.61 | 11.17 | 10.63 | 10.53 | 5.72E-10 |
| mglC | APL_1418 | Galactoside transport system permease protein | 10.79 | 10.35 | 9.69 | 9.47 | 6.69E-10 |
| tktA | APL_0983 | Transketolase 2 | 12.92 | 12.75 | 12.12 | 11.86 | 8.84E-10 |

| | | | | | | | |
|---|---|---|---|---|---|---|---|
| APL_0141 | APL_0141 | Uncharacterized transporter | 9.82 | 9.44 | 9.12 | 8.82 | 1.04E-09 |
| APL_1919 | APL_1919 | Putative uncharacterized protein | 11.89 | 11.41 | 10.56 | 10.13 | 1.12E-09 |
| malQ | APL_1240 | 4-alpha-glucanotransferase | 12.57 | 11.99 | 11.16 | 10.62 | 1.22E-09 |
| yvfF | APL_1030 | Polysaccharide pyruvyl transferase | 9.48 | 9.05 | 8.43 | 8.18 | 1.40E-09 |
| *Coenzyme transport and metabolism* | | | | | | | |
| chuW | APL_1523 | Coproporphyrinogen III oxidase | 11.28 | 10.7 | 9.86 | 9.36 | 1.45E-13 |
| hemH | APL_1937 | Ferrochelatase | 9.82 | 8.97 | 8.5 | 8.17 | 6.50E-13 |
| menC | APL_0353 | O-succinylbenzoate synthase | 9.12 | 8.58 | 8.2 | 7.99 | 1.09E-12 |
| dfp | APL_1969 | Coenzyme A biosynthesis bifunctional protein | 9.82 | 9.13 | 8.78 | 8.09 | 3.96E-12 |
| serC | APL_0702 | Phosphoserine aminotransferase | 9.95 | 9.58 | 9.32 | 9.07 | 4.60E-12 |
| rraA | APL_1901 | Ribonuclease activity regulator protein | 11.37 | 10.98 | 10.43 | 9.9 | 5.86E-11 |
| rimK | APL_0484 | Ribosomal protein S6 modification protein | 10.63 | 10.36 | 9.85 | 9.58 | 1.64E-09 |
| *Lipid transport and metabolism* | | | | | | | |
| accA | APL_1486 | Acetyl-coenzyme A carboxylase carboxyl transferase | 11.77 | 11.18 | 10.85 | 10.36 | 4.68E-14 |
| APL_1413 | APL_1413 | Putative long-chain-fatty-acid--CoA ligase | 12.48 | 12.22 | 11.7 | 11.44 | 1.02E-09 |
| fadJ | APL_0888 | Fatty oxidation complex, alpha subunit | 10.89 | 10.4 | 9.91 | 9.46 | 1.09E-09 |
| *Translation* | | | | | | | |
| cysS | APL_0913 | Translation, ribosomal structure and biogenesis | 11.5 | 11.21 | 10.69 | 10.4 | 2.76E-13 |
| rsmB | APL_1560 | Ribosomal RNA small subunit methyltransferase B | 9.89 | 9.36 | 9.03 | 8.55 | 6.27E-13 |
| selB | APL_1561 | Selenocysteine-specific elongation factor | 10.72 | 10.46 | 9.87 | 9.65 | 5.38E-11 |
| poxA | APL_1530 | PoxB regulator | 10.98 | 10.44 | 9.74 | 9.09 | 1.16E-10 |
| rluC | APL_1877 | Pseudouridine synthase | 12.77 | 12.47 | 11.95 | 11.68 | 3.11E-10 |
| APL_1210 | APL_1210 | Putative uncharacterized protein | 10.56 | 9.98 | 9.23 | 8.7 | 3.71E-10 |
| gltX | APL_1275 | Glutamyl-tRNA synthetase | 11.8 | 11.69 | 10.94 | 10.65 | 1.67E-09 |
| *Transcription* | | | | | | | |
| ulaR | APL_1702 | HTH-type transcriptional regulator | 11.3 | 10.74 | 9.68 | 9.36 | 4.27E-13 |
| APL_0706 | APL_0706 | Putative HTH-type transcriptional regulator | 12.48 | 12.02 | 10.65 | 10.49 | 2.62E-12 |
| nusA | APL_0638 | Transcription elongation protein | 14.22 | 14.52 | 14.62 | 14.8 | 4.75E-11 |
| cspD | APL_0725 | Cold shock-like protein | 9.43 | 8.7 | 8.14 | 7.77 | 1.22E-10 |
| *Replication, recombination and repair* | | | | | | | |
| radC | APL_1970 | DNA repair protein | 9.37 | 8.16 | 7.85 | 7.3 | 1.04E-15 |
| APL_1347 | APL_1347 | Putative methylase | 10.24 | 9.76 | 9.32 | 9.09 | 5.21E-15 |
| mfd | APL_1339 | transcription-repair coupling factor | 11.39 | 10.92 | 10.24 | 9.93 | 5.94E-14 |
| tagL | APL_1931 | 3-methyladenine-DNA glycosidase | 9.85 | 9.29 | 8.87 | 8.43 | 5.96E-13 |
| mutL | APL_1958 | DNA mismatch repair protein | 9.71 | 9.29 | 8.87 | 8.47 | 8.08E-12 |
| topB2 | APL_0744 | DNA Topoisomerase III | 10.76 | 10.48 | 9.89 | 9.7 | 8.19E-11 |
| recC | APL_1884 | Exodeoxyribonuclease V gamma chain | 10.78 | 10.17 | 9.56 | 9.19 | 1.54E-10 |
| nth | APL_0886 | endonuclease III | 10.86 | 10.51 | 9.93 | 9.66 | 1.81E-10 |
| comE1 | APL_1406 | DNA uptake protein/ Fibronectin binding | 13 | 12.33 | 11.56 | 11.19 | 8.69E-10 |
| *Cell wall/membrane biogenesis* | | | | | | | |
| wecD | APL_1550 | Putative TDP-D-fucosamine acetyltransferase | 9.71 | 9.05 | 8.58 | 8 | 6.51E-16 |
| lrgB | APL_0779 | Putative effector of murein hydrolase | 10.63 | 9.83 | 9.16 | 8.4 | 7.16E-15 |
| wecC | APL_1551 | UDP-N-acetyl-D-mannosamine dehydrogenase | 10.45 | 10.03 | 9.33 | 8.69 | 8.68E-15 |
| murI | APL_1841 | Glutamate racemase | 11.08 | 10.65 | 10.13 | 9.77 | 9.54E-13 |
| ompW | APL_1086 | Outer membrane protein W | 12.07 | 11.88 | 10.8 | 10.17 | 7.01E-12 |

| | | | | | | | |
|---|---|---|---|---|---|---|---|
| wecE | APL_1549 | TDP-4-keto-6-deoxy-D-glucose transaminase | 10.13 | 9.71 | 8.98 | 8.45 | 1.07E-11 |
| rsmH | APL_0010 | Ribosomal RNA small subunit methyltransferase H | 11.39 | 11.18 | 10.71 | 10.45 | 1.30E-11 |
| wecB | APL_1552 | UDP-N-acetylglucosamine 2-epimerase | 10.63 | 10.28 | 9.37 | 8.91 | 1.33E-11 |
| APL_0234 | APL_0234 | 23S rRNA pseudouridine synthase D | 11.92 | 11.36 | 10.55 | 10.33 | 1.53E-11 |
| murD | APL_0016 | UDP-N-acetylmuramoylalanine-D-glutamate ligase | 11.63 | 11.32 | 10.69 | 10.35 | 3.26E-11 |
| dgkA | APL_0768 | Diacylglycerol kinase | 11.38 | 10.9 | 10.33 | 10.04 | 3.99E-11 |
| macA | APL_0391 | Probable macrolide-specific efflux protein | 11.74 | 11.44 | 10.52 | 10.49 | 4.45E-10 |
| kdsB | APL_0085 | 3-deoxy-manno-octulosonate cytidylyltransferase | 10.82 | 10.27 | 9.5 | 9.14 | 8.10E-10 |
| *Cell motility* | | | | | | | |
| apfA | APL_0880 | Possible prepilin peptidase dependent protein D | 12.14 | 11.28 | 11.13 | 10.61 | 2.56E-10 |
| apfB/hofB | APL_0879 | Fimbrial biogenesis protein | 11.57 | 10.64 | 10.01 | 9.31 | 1.50E-09 |
| *Posttranslational modification, protein turnover, chaperones* | | | | | | | |
| comM | APL_1747 | Competence protein comM | 9.49 | 8.98 | 8.9 | 8.53 | 4.89E-16 |
| dipZ | APL_1359 | Thiol:disulfide interchange protein DsbD | 9.34 | 9.01 | 8.69 | 8.44 | 1.35E-15 |
| gst | APL_1524 | Glutathione S-transferase | 11.14 | 10.73 | 10.15 | 9.67 | 4.51E-15 |
| ureE | APL_1614 | Urease accessory protein ureE | 9.36 | 8.65 | 8.11 | 7.45 | 2.58E-14 |
| nrfG | APJL_1067 | Formate-dependent nitrite reductase complex | 10.98 | 10.36 | 9.35 | 8.79 | 4.79E-14 |
| nrfF | APJL_1068 | Formate-dependent nitrite reductase complex | 9.39 | 9.08 | 8.66 | 8.33 | 8.67E-13 |
| hypF | APL_1330 | Hypothetical carbamoyltransferase | 10.59 | 9.95 | 9.18 | 8.97 | 1.39E-11 |
| ureD | APL_1611 | Urease accessory protein ureD | 11.01 | 10.32 | 9.28 | 8.82 | 3.46E-11 |
| tpx | APL_1489 | Thiol peroxidase | 11.31 | 10.63 | 10.18 | 9.38 | 6.54E-11 |
| hypD | APL_1328 | Hydrogenase isoenzymes formation protein | 11.25 | 11.04 | 10.07 | 9.61 | 1.11E-10 |
| tusA/sirA | APL_0092 | Sulfurtransferase tusA homolog | 10.32 | 9.53 | 8.71 | 8.19 | 2.21E-10 |
| smpB | APL_0867 | SsrA-binding protein | 13.1 | 12.93 | 12.31 | 12.14 | 2.70E-10 |
| nrfE | APL_1052 | Cytochrome c-type bioproteinsis protein | 11.23 | 11.2 | 10.43 | 9.93 | 2.94E-10 |
| ureG | APL_1612 | urease accessory protein | 9.45 | 8.84 | 8.89 | 8.36 | 3.18E-10 |
| ptrA | APL_1883 | Protease III | 11.31 | 10.77 | 10.14 | 9.86 | 3.98E-10 |
| ppiB | APL_0914 | Peptidyl-prolyl cis-trans isomerase | 11.46 | 10.73 | 10.26 | 10.08 | 5.39E-10 |
| sppA | APL_1268 | Protease 4 | 11.05 | 10.57 | 9.84 | 9.53 | 8.00E-10 |
| dsbE2/nrfX | APL_1051 | Thiol:disulfide interchange protein | 11.37 | 11.16 | 10.07 | 9.5 | 1.04E-09 |
| *Inorganic ion transport and metabolism* | | | | | | | |
| ccp | APL_1379 | Cytochrome c peroxidase | 11.53 | 10.56 | 9.88 | 9.32 | 3.07E-17 |
| afuA | APL_1446 | ABC-type Fe3+ transport system | 12.45 | 12.06 | 10.93 | 10.25 | 1.01E-16 |
| eriC | APL_0674 | Chloride channel protein | 10.3 | 10.05 | 9.86 | 9.52 | 4.52E-15 |
| APL_1285 | APL_1285 | Rhodanese-related sulfurtransferase | 12.8 | 12.46 | 11.4 | 11.09 | 1.16E-13 |
| kefBC | APL_1053 | Glutathione-regulated potassium-efflux system protein | 11.8 | 11.64 | 10.92 | 10.69 | 4.87E-13 |
| ywbN | APL_0669 | Putative iron dependent peroxidase | 11.22 | 10.2 | 9.79 | 9.28 | 1.82E-12 |
| APL_0668 | APL_0668 | Possible periplasmic iron (Fe) transport lipoprotein | 10.83 | 9.7 | 9.36 | 9.09 | 3.89E-12 |
| plpA/hplA | APL_0910 | Outer membrane lipoprotein | 10.76 | 10.39 | 9.61 | 9.44 | 4.13E-12 |
| APL_1162 | APL_1162 | Predicted iron-dependent peroxidase | 9.74 | 9.45 | 9.21 | 8.96 | 1.02E-11 |
| afuB/fbpB | APL_1447 | Ferric transport system permease protein | 11.95 | 11.77 | 10.52 | 9.74 | 3.19E-11 |
| citT | APJL_1176 | CitT di-and tricarboxylate transporter | 9.67 | 9.37 | 9.04 | 8.6 | 3.82E-11 |
| fhuC | APL_2013 | Ferrichrome transport ATP-binding protein | 11.39 | 11.05 | 10.28 | 10.17 | 4.59E-11 |
| nrfA | APL_0100 | Cytochrome c-552 | 13.64 | 13.56 | 12.22 | 12 | 1.10E-10 |
| hmuV/fecE | APL_1793 | Hemin ABC superfamily ATP binding cassette transporter | 9.16 | 8.88 | 8.33 | 7.75 | 2.15E-10 |

| | | | | | | | |
|---|---|---|---|---|---|---|---|
| nhaB | APL_0322 | Na(+)/H(+) antiporter | 11.32 | 11.08 | 10.04 | 9.73 | 2.37E-10 |
| nrfB | APL_0101 | Cytochrome c-type protein | 12.54 | 12.4 | 10.81 | 10.43 | 2.60E-10 |
| APL_1264 | APL_1264 | Putative cation transport ATPase | 9.68 | 9.04 | 8.42 | 8.18 | 3.32E-10 |
| tehA | APL_1212 | Tellurite resistance protein and related permease | 11.27 | 10.98 | 10.44 | 10.11 | 3.81E-10 |
| copA | APL_1265 | Copper-transporting P-type ATPase | 9.76 | 9.36 | 8.91 | 8.7 | 1.66E-09 |
| *Secondary metabolites biosynthesis, transport and catabolism* | | | | | | | |
| APL_1423 | APL_1423 | Putative uncharacterized protein | 10.89 | 10.42 | 10.07 | 9.57 | 1.47E-17 |
| sufI1 | APL_1487 | Protein SufI precursor | 10.68 | 10.15 | 9.53 | 9 | 8.12E-12 |
| sufI | APL_2035 | Copper-containing nitrite reductase | 9.51 | 9.32 | 9.09 | 8.99 | 2.29E-11 |
| *Intracellular trafficking and secretion* | | | | | | | |
| hofQ | APL_0200 | Type II secretory pathway, component HofQ | 10.54 | 9.57 | 9.04 | 8.53 | 8.87E-15 |
| tatA | APL_1985 | Sec-independent protein translocase protein | 12.87 | 12.54 | 11.85 | 11.94 | 1.36E-10 |
| ftsY | APL_1346 | Cell division protein | 11.1 | 10.77 | 10.02 | 9.78 | 1.08E-09 |
| *General function prediction only* | | | | | | | |
| APL_1213 | APL_1213 | Putative 2-deoxyglucose-6-phosphatase | 10.94 | 10.29 | 9.5 | 8.94 | 4.89E-16 |
| hpaC | APL_1349 | 4-hydroxyphenylacetic acid hydroxylase | 12.56 | 12.1 | 11.25 | 10.5 | 3.21E-14 |
| ompP4 | APL_0389 | Lipoprotein E | 12.46 | 11.71 | 10.77 | 9.98 | 4.29E-14 |
| torD | APL_1797 | Chaperone protein | 9.65 | 9.15 | 8.75 | 8.52 | 7.59E-14 |
| rssA | APL_1996 | Putative phosphoesterase | 10.58 | 9.89 | 9.2 | 8.72 | 4.59E-13 |
| dcuB1 | APL_1532 | Anaerobic C4-dicarboxylate transporter | 12.99 | 12.82 | 11.95 | 11.67 | 1.08E-12 |
| APL_0392 | APL_0392 | Putative uncharacterized protein | 11.45 | 11.11 | 10.44 | 10.05 | 1.76E-12 |
| APL_1522 | APL_1522 | Nucleoside-diphosphate-sugarepimerase | 10.88 | 10.54 | 10.15 | 9.69 | 2.17E-12 |
| sufE | APL_1281 | Protein probably involved in Fe-S center assembly | 9.99 | 9.27 | 8.56 | 8.11 | 2.40E-12 |
| APL_0390 | APL_0390 | Sodium Bile acid symporter family protein | 10.57 | 10.32 | 9.96 | 9.74 | 6.45E-12 |
| wzxE | APL_1548 | Lipopolysaccharide biosynthesis protein | 11.09 | 10.76 | 10.31 | 9.95 | 1.21E-11 |
| dcuB2 | APL_1316 | Anaerobic C4-dicarboxylate transporter DcuB | 12.63 | 12.44 | 11.72 | 11.29 | 1.31E-11 |
| APL_0047 | APL_0047 | Diadenosine tetraphosphatase | 11.24 | 10.94 | 10.45 | 10.03 | 1.39E-11 |
| APL_0851 | APL_0851 | ABC transporter, ATP-binding subunit | 10.9 | 10.53 | 9.31 | 9.14 | 1.48E-11 |
| APL_1412 | APL_1412 | Putative oxidoreductase | 11.04 | 10.44 | 10.18 | 10.03 | 1.74E-11 |
| APL_0885 | APL_0885 | Permease with 5 transmembrane domains | 10.42 | 10.14 | 9.73 | 9.1 | 3.16E-11 |
| APL_1355 | APL_1355 | Phospholipid-binding protein | 10.82 | 10.04 | 9.32 | 8.74 | 3.41E-11 |
| APL_1997 | APL_1997 | NADPH-dependent FMN reductase | 12.17 | 11.45 | 10.77 | 10.38 | 8.23E-11 |
| ulaG | APL_1701 | L-ascorbate-6-phosphate lactonase | 12.45 | 11.92 | 11.37 | 11.03 | 9.03E-11 |
| APL_1044 | APL_1044 | Permease | 9.61 | 9.08 | 8.65 | 8.34 | 2.04E-10 |
| APL_0440 | APL_0440 | Putative uncharacterized protein | 9.39 | 9.03 | 8.67 | 8.09 | 2.10E-10 |
| APL_0966 | APL_0966 | Putative transport protein | 10.72 | 10.71 | 10.4 | 10.07 | 5.12E-10 |
| APL_1180 | APL_1180 | Hypothetical oxidoreductase | 11.58 | 11.24 | 10.6 | 10.21 | 6.88E-10 |
| APL_0866 | APL_0866 | Putative ATPase | 10.19 | 9.54 | 8.99 | 8.53 | 7.38E-10 |
| APL_0051 | APL_0051 | 3-deoxy-D-manno-octulosonate 8-phosphate phosphatase | 10.35 | 9.79 | 9.37 | 9.03 | 8.81E-10 |
| APL_1294 | APL_1294 | Putative uncharacterized protein | 10.37 | 10.07 | 9.58 | 9.25 | 1.28E-09 |
| *Function unknown* | | | | | | | |
| ulaA | APL_1714 | Ascorbate-specific permease IIC component | 11.35 | 10.67 | 9.57 | 8.48 | 6.02E-18 |
| APL_0031 | APL_0031 | Integral membrane protein | 9.46 | 8.85 | 8.16 | 7.64 | 7.69E-16 |
| ulaA (sgaT) | APL_1700 | PTS system ascorbate-specific transporter subunits IICB | 10.63 | 9.64 | 9.48 | 9.2 | 1.12E-15 |
| dhaM | APL_0081 | PTS-dependent dihydroxyacetone kinase | 11.49 | 11.14 | 10.31 | 9.94 | 1.07E-14 |

| | | | | | | | |
|---|---|---|---|---|---|---|---|
| APL_1284 | APL_1284 | Putative DNA-binding protein | 11.14 | 10.63 | 9.96 | 9.65 | 2.81E-14 |
| APL_1605 | APL_1605 | UPF0597 protein | 9.37 | 9.04 | 8.84 | 8.51 | 3.18E-14 |
| APL_1713 | APL_1713 | Putative oligopeptide transporter | 10.63 | 10.2 | 9.66 | 8.87 | 4.68E-14 |
| APL_1589 | APL_1589 | Putative uncharacterized protein | 10.01 | 9.68 | 9.39 | 9.25 | 8.99E-14 |
| APL_1935 | APL_1935 | Putative uncharacterized protein | 10.85 | 10.48 | 9.46 | 8.51 | 3.16E-13 |
| APL_0215 | APL_0215 | Putative uncharacterized protein | 10.83 | 9.69 | 9 | 8.68 | 3.66E-13 |
| APL_1360 | APL_1360 | Putative uncharacterized protein | 11.24 | 10.71 | 10.12 | 9.76 | 2.28E-12 |
| APL_0216 | APL_0216 | Putative uncharacterized protein | 9.74 | 8.67 | 8.14 | 7.91 | 2.45E-12 |
| comB | APL_0197 | Putative uncharacterized protein | 10.65 | 9.77 | 9.34 | 9.08 | 3.53E-12 |
| APL_1348 | APL_1348 | Ds-DNA-mimic protein | 11.8 | 11.42 | 10.43 | 9.76 | 7.08E-12 |
| APL_1211 | APL_1211 | Putative uncharacterized protein | 9.61 | 9.25 | 8.75 | 8.55 | 9.24E-12 |
| APL_0431 | APL_0431 | Putative uncharacterized protein | 11.77 | 11.39 | 10.53 | 10.22 | 2.11E-11 |
| APL_1946 | APL_1946 | Integral membrane protein | 9.26 | 8.34 | 7.93 | 7.34 | 3.06E-11 |
| APL_1966 | APL_1966 | Putative uncharacterized protein | 9.8 | 9.43 | 9.01 | 8.59 | 4.67E-11 |
| APL_1610 | APL_1610 | Putative uncharacterized protein | 10.95 | 10.15 | 9.09 | 8.53 | 7.94E-11 |
| APL_0709 | APL_0709 | UPF0265 protein | 11.18 | 10.31 | 9.6 | 9.07 | 8.06E-11 |
| yhaH | APL_0032 | Putative uncharacterized protein yhaH | 11.24 | 10.78 | 9.72 | 9.21 | 1.39E-10 |
| mraZ | APL_0009 | Protein mraZ | 13.77 | 13.69 | 13.22 | 12.95 | 1.75E-10 |
| APL_1697 | APL_1697 | Putative uncharacterized protein | 9.44 | 8.96 | 8.75 | 8.58 | 2.26E-10 |
| APL_0111 | APL_0111 | Putative uncharacterized protein | 10.69 | 10.21 | 10.01 | 9.72 | 2.27E-10 |
| APL_0949 | APL_0949 | 8-amino-7-oxononanoate synthase | 10.76 | 10.34 | 9.62 | 9.2 | 3.62E-10 |
| APL_1203 | APL_1203 | Putative uncharacterized protein | 11.05 | 10.61 | 9.78 | 9.35 | 5.75E-10 |
| rimP | APL_0637 | Ribosome maturation factor | 13.61 | 13.98 | 14.11 | 14.24 | 6.37E-10 |
| APL_1894 | APL_1894 | Putative uncharacterized protein | 9.59 | 9.28 | 8.18 | 7.28 | 6.81E-10 |
| APL_0179 | APL_0179 | Putative uncharacterized protein | 9.41 | 8.87 | 8.53 | 8.27 | 7.74E-10 |
| APL_0373 | APL_0373 | Putative uncharacterized protein | 10.69 | 10.04 | 9.41 | 9.18 | 1.11E-09 |
| APL_2012 | APL_2012 | Putative uncharacterized protein | 9.79 | 9.42 | 8.99 | 8.58 | 1.52E-09 |
| *Not in COGs* | | | | | | | |
| 23S rRNA | APL_2115 | 23S ribosomal RNA | 12.51 | 13.16 | 13.57 | 13.87 | 2.09E-14 |
| 16S rRNA | APL_2109 | 16S ribosomal RNA | 13.58 | 14.17 | 14.49 | 14.76 | 6.01E-13 |
| APJL_0401 | APJL_0401 | Putative uncharacterized protein | 10.81 | 10.04 | 8.88 | 7.97 | 1.30E-11 |
| APL_2056 | APL_2056 | tRNA-Leu | 10.36 | 9.62 | 8.82 | 7.96 | 1.38E-11 |
| APJL_1298 | APJL_1298 | Putative uncharacterized protein | 13.68 | 13.29 | 12.52 | 12.31 | 8.37E-11 |
| APL_1102 | APL_1102 | Putative uncharacterized protein | 10.57 | 9.88 | 9.05 | 8.63 | 1.31E-09 |

Genes are ordered according to Clusters of Orthologous Groups of proteins (COGs).

# Locus numbers from *A. pleuropneumoniae* serotype 5 (L20) or alternatively, if a gene could not be identified in serotype 5, then from *A. pleuropneumoniae* serotype 3.

These locus numbers were applied to facilitate comparison with othe studies of *A. pleuropneumoniae* expression.

* $Log_2$ mean values of absolute expression (n = 75).

Grey bars: genes likewise found to be differentially expressed in other expression studies of *A. pleuropneumoniae* or *H. influenzae* (for details see Table S5).
