# Supplementary material for: Transcriptional Portrait of Actinobacillus pleuropneumoniae during Acute Disease - Potential Strategies for Survival and Persistence in the Host
Source: PLoS One. 2012 Apr 17;7(4):e35549. doi: 10.1371/journal.pone.0035549 (PMC3328466; doi:10.1371/journal.pone.0035549)
Supplement: Table S4 — The most highly and constitutively expressed genes of A. pleuropneumoniae during the first 48 h post experimental challenge. (PDF) [file pone.0035549.s005.pdf]

Table S4. The most highly and constitutively expressed genes of *A. pleuropneumoniae* during the first 48 h post experimental challenge

Gene designation	Locus no.[#]	Annotation	Mean Log_2 expression ($Log_2 \geq 13$)*	Log_2 STDV**
Energy production and conversion				
nqrE	APL_0154	Na(+)-translocating NADH-quinone reductase subunit E	13.89	0.41
ackA	APL_0645	Acetate kinase	13.80	0.42
cydB	APL_0298	Cytochrome D ubiquinol oxidase, subunit II	13.79	0.39
atpA	APL_1648	ATP synthase subunit alpha	13.77	0.48
nqrC	APL_0152	Na(+)-translocating NADH-quinone reductase subunit C	13.77	0.38
nqrF	APL_0155	Na(+)-translocating NADH-quinone reductase subunit F	13.76	0.34
atpC	APL_1645	ATP synthase epsilon chain	13.36	0.49
pflB	APL_1036	Formate acetyltransferase	13.28	0.47
cydA	APL_0297	Cytochrome D ubiquinol oxidase, subunit I	13.27	0.45
nqrA	APL_0150	NADH:ubiquinone oxidoreductase, Na(+)-translocating, subunit A	13.27	0.41
aceE	APL_0773	pyruvate dehydrogenase E1 component	13.12	0.45
nqrB	APL_0151	Na(+)-translocating NADH-quinone reductase subunit B	13.00	0.48
atpD	APL_1646	ATP synthase subunit beta	13.00	0.45
Cell cycle control, mitosis and meiosis				
ftsZ	APL_0023	Cell division protein	13.64	0.32
mreB	APL_0435	Rod shape-determining protein	13.12	0.26
Amino acid transport and metabolism				
ilvC	APL_1853	Ketol-acid reductoisomerase	14.67	0.35
argD1	APL_1974	Diaminobutyrate--2-oxoglutarate aminotransferase	13.66	0.37
sstT	APL_0767	Serine/threonine transporter	13.19	0.40
aroB	APL_0193	3-dehydroquinate synthase	13.13	0.38
Nucleotide transport and metabolism				
cmkA	APL_0741	Cytidylate kinase	13.24	0.35
Carbohydrate transport and metabolism				
ptsL	APL_1323	Phosphoenolpyruvate-protein phosphotransferase	14.31	0.29
gapA	APL_0434	Glyceraldehyde-3-phosphate dehydrogenase	14.05	0.48
pykA	APL_0187	Pyrovate kinase	14.05	0.33
crr	APL_1324	Glucose-specific phosphotransferase enzyme IIA component	14.02	0.27
APL_0737	APL_0737	Uncharacaterized protein	13.68	0.40
ptsN	APL_0335	PTS system, nitrogen regulatory IIA-like protein	13.34	0.43
ptnD	APL_1393	PTS system, mannose-specific IID component	13.23	0.48
fba	APL_1250	Fructose-bisphosphate aldolase	13.20	0.46
Coenzyme transport and metabolism				
hemX	APL_1008	Putative uroporphyrin-III C-methyltransferase	13.45	0.42
dxs	APL_0207	1-deoxy-D-xylulose-5-phosphate synthase	13.38	0.40
hemC	APL_1010	Porphobilinogen deaminase	13.27	0.37
Lipid transport and metabolism				
plsX	APL_1385	Phosphate acyltransferase	13.79	0.40
accD	APL_0631	Acetyl-coenzyme A carboxylase carboxyl transferase subunit beta	13.26	0.49
APL_0971	APL_0971	Putative acyl CoA thioester hydrolase	13.12	0.36
Translation				
rplM	APL_0600	50S ribosomal protein L13	15.21	0.20
rpmC	APL_1768	50S ribosomal protein L29	15.03	0.25
rplX	APL_1770	50S ribosomal protein L24	15.00	0.29
rplD	APL_1761	50S ribosomal protein L4	14.99	0.21
rplW	APL_1762	50S ribosomal protein L23	14.89	0.28
rpsA	APL_0740	30S ribosomal protein S1	14.82	0.25
rpsE	APL_1776	30S Ribosomal protein S5	14.82	0.25
rpsC	APL_1766	30S ribosomal protein S3	14.75	0.22
rpmD	APL_1777	50S Ribosomal protein L30	14.74	0.32
rplC	APL_1760	50S ribosomal protein L3	14.74	0.29
rplF	APL_1774	50S ribosomal protein L6	14.70	0.27
rpmF	APL_1386	50S ribosomal protein L32	14.68	0.38
infC	APL_0223	Translation initiation factor IF-3	14.68	0.24
rpmA	APL_2000	50S ribosomal protein L27	14.67	0.32
rplB	APL_1763	50S ribosomal protein L2	14.66	0.26
rplP	APL_1767	50S ribosomal protein L16	14.62	0.29
rpsK	APL_1782	30S ribosomal protein S11	14.60	0.27
rplA	APL_1719	50S ribosomal protein L1	14.59	0.26
rplS	APL_1789	50S ribosomal protein L19	14.53	0.44
rplJ	APL_1720	50S ribosomal protein L10	14.53	0.24

rpsL	APL_0601	30S ribosomal protein S9	14.49	0.27
rplR	APL_1775	50S ribosomal protein L18	14.48	0.31
rplV	APL_1765	50S ribosomal protein L22	14.48	0.31
fusA	APL_1399	elongation factor G	14.48	0.29
rplO	APL_1778	50S ribosomal protein L15	14.47	0.41
rpmL	APL_0224	50S ribosomal protein L35	14.46	0.42
infB	APL_0639	Translation initiation factor IF-2	14.45	0.46
trmD	APL_1788	tRNA (guanine-N(1)-)-methyltransferase	14.45	0.47
rlpT	APL_0225	50S ribosomal protein L20	14.43	0.44
rpsD	APL_1783	30S ribosomal protein S4	14.41	0.27
rplN	APL_1769a	50S ribosomal protein L14	14.37	0.39
tufB	APL_1398	Elongation factor Tu	14.33	0.24
rpmJ1	APL_1780	50S ribosomal protein L36 1	14.29	0.38
rplL	APL_1721	50S ribosomal protein L7/L12	14.28	0.33
rplI	APL_1169	50S ribosomal protein L9	14.25	0.44
rpsG	APL_1400	30S ribosomal protein S7	14.24	0.36
rpsN	APL_1772	30S ribosomal protein S14	14.19	0.35
rplE	APL_1771	50S ribosomal protein L5	14.18	0.38
rpsH	APL_1773	30S ribosomal protein S8	14.18	0.39
rpsM	APL_1781	30S ribosomal protein S13	14.13	0.34
rpsJ	APL_1759	30S ribosomal protein S10	14.12	0.43
rpsQ	APL_1769	30S ribosomal protein S17	14.08	0.39
rpsF	APL_1171	30S ribosomal protein S6	14.03	0.38
rpsB	APL_0566	30S ribosomal protein S2	13.87	0.37
tsf	APL_0567	Elongation factor Ts	13.85	0.37
rplQ	APL_1785	50S ribosomal protein L17	13.73	0.37
rplK	APL_1718	50S ribosomal protein L11	13.06	0.42
Transcription				
rpoA	APL_1784	DNA-directed RNA polymerase subunit alpha	14.69	0.28
nusA	APL_0638	Transcription termination factor	14.54	0.31
rpoD	APL_1475	RNA polymerase sigma factor	13.53	0.39
rho	APL_0247	Transcription termination factor	13.13	0.45
nusG	APL_1717	Transcription antitermination protein	13.12	0.39
APL_0932	APL_0932	Putative HTH-type transcriptional regulator	13.02	0.47
Replication, recombination and repair				
priB	APL_1170	Primosomal replication protein n	14.46	0.25
ihfB	APL_0739	Integration host factor subunit beta	14.29	0.34
gyrB	APL_0821	DNA gyrase subunit B	13.28	0.34
deaD	APL_0575	cold-shock DEAD box protein A-like protein	13.10	0.36
Cell wall/membrane biogenesis				
ompA	APL_1852	Outer membrane protein P5 precursor (OMP P5)	14.53	0.28
palA	APL_0304	Outer membrane protein	14.31	0.33
ompA	APL_1421	Outer membrane protein P5 precursor	14.07	0.25
csgG	APL_0220	Putative lipoprotein	14.05	0.41
APL_1597	APL_1597	Possible rare lipoprotein A	13.63	0.49
acrA	APL_0586	Putative RND efflux membrane fusion protein	13.37	0.38
nlpC	APL_0359	Putative lipoprotein	13.27	0.49
tolA	APL_0302	Cell envelope integrity inner membrane protein	13.26	0.38
mltA	APL_0816	Murein transglycosylase A	13.23	0.41
lpxC	APL_0024	UDP-3-O-[3-hydroxymyristoyl] N-acetylglucosamine deacetylase	13.21	0.40
prc	APL_0120	Carboxy-terminal protease	13.14	0.41
ftsQ	APL_0021	Cell division protein	13.13	0.46
mltC	APL_1741	Membrane-bound lytic murein transglycosylase C	13.06	0.42
glmS	APL_1631	Glucosamine-fructose-6-phosphate aminotransferase	13.03	0.47
Posttranslational modification, protein turnover, chaperones				
dnaK	APL_1906	Chaperone protein	14.24	0.44
surA	APL_0400	Survival SurA-like protein	13.65	0.43
degS	APL_0742	Protease DegS precursor	13.65	0.38
dnaJ	APL_1905	Chaperone protein	13.39	0.49
djlA	APL_0306	DnaJ-like protein	13.31	0.38
Inorganic ion transport and metabolism				
fur	APL_1218	Ferric uptake regulator	13.40	0.46
APL_1508	APL_1508	Rhodanese-related sulfurtransferase	13.39	0.45
APL_0719	APL_0719	Putative phosphate permease	13.06	0.33
Secondary metabolites biosynthesis, transport and catabolism				
apxIIIA	APJL_1346	RTX-III toxin determinant A	13.95	0.37
APL_1291	APL_1291	Probable permease	13.14	0.47

Signal transduction mechanisms				
typA	APL_0053	GTP-binding protein	13.54	0.38
Intracellular trafficking and secretion				
secY	APL_1779	Preprotein translocase subunit	14.06	0.42
tatB	APL_1986	Sec-independent protein translocase-like protein	13.64	0.38
yajC	APL_1066	Preprotein translocase subunit	13.49	0.37
secD	APL_1067	Protein-export membrane protein	13.28	0.37
secB	APL_1509	Protein-export protein	13.12	0.47
secF	APL_1068	Protein-export membrane protein	13.10	0.41
Defense mechanisms				
apxID	APL_1442	RTX-I toxin secretion component	13.15	0.46
General function prediction only				
nlpI	APL_0576	Lipoprotein NlpI-like	14.04	0.32
APL_1121	APL_1121	Putative lipoprotein	13.53	0.41
sspB	APL_0657	ClpXP protease specificity-enhancing factor/Stringent starvation protein B	13.16	0.49
trmB	APL_1383	tRNA (guanine-N(7)-)-methyltransferase)	13.13	0.45
APL_1896	APL_1896	Putative uncharacterized protein	13.09	0.38
Function unknown				
APL_0221	APL_0221	Putative lipoprotein, periplasmic protein	14.46	0.49
rimP	APL_0637	Ribosome maturation factor	13.98	0.39
APL_0121	APL_0121	Uncharacaterized protein	13.59	0.37
yhaH	APL_0032	Integral membrane protein	13.41	0.42
APL_1790	APL_1790	Exodeoxyribonuclease VII large subunit	13.39	0.41
APL_0116	APL_0116	Putative DNA polymerase III subunit delta	13.20	0.45

Genes are ordered according to Clusters of Orthologous Groups of proteins (COGs).
# Locus numbers from *A. pleuropneumoniae* serotype 5 (L20).
* Log_2 mean values of absolute expression values of time points 6h, 12h, 24h and 48h p.i. (n = 75).
** Log_2 standard deviation of absolute expression values (n = 75).
Grey bars: genes likewise found to be differentially expressed in other expression studies of *A. pleuropneumoniae* or *H. influenzae* (for details see Table S5).
