# Supplementary material for: Transcriptional Portrait of Actinobacillus pleuropneumoniae during Acute Disease - Potential Strategies for Survival and Persistence in the Host
Source: PLoS One. 2012 Apr 17;7(4):e35549. doi: 10.1371/journal.pone.0035549 (PMC3328466; doi:10.1371/journal.pone.0035549)
Supplement: Table S5 — Genes found to be differentially expressed in the present and other expression studies of A. pleuropneumoniae or H. influenzae. (PDF) [file pone.0035549.s006.pdf]

Table S5. Genes found to be differentially expressed in the present and other expression studies of *A. pleuropneumoniae* or *H. influenzae.*

**Gene designation**	**Locus no.**	**Annotation**	**This study**	**I**	**II**	**III**	**IV**	**V**	**VI**	**VII**
Energy production and conversion										
nrfC	APL_0102	Nitrate reductase	Diff expr		up					
glpK	APL_0375	Glycerol kinase	Diff expr	up						
maeB	APL_0486	NADP-dependent malic enzyme (NADP-ME)	Diff expr			down				
nfnB	APL_0607	Putative NAD(P)H nitroreductase	Diff expr	down						
torZ	APL_0688	Trimethylamine-N-oxide reductase precursor	Diff expr			down				
lpdA	APL_0771	Dihydrolipoyl dehydrogenase	Diff expr	down		down				STM*
dcuC	APL_0870	Putative C4-dicarboxylate transporter	Diff expr	up		up			up	
hyaA	APL_1331	Hydrogenase 2 small subunit	Diff expr		up	up				
hybB	APL_1333	Putative Ni/Fe-hydrogenase 2 b-type cytochrome subunit	Diff expr	up		up				
hyaB	APL_1334	Hydrogenase-2 large chain	Diff expr		up					
dmsA	APL_1674	Anaerobic dimethyl sulfoxide reductase chain A precursor	Diff expr		up		SCOTS			
atpD	APL_1646	ATP synthase subunit beta	High expr					HITS		
nqrB	APL_0151	Na(+)-translocating NADH-quinone reductase subunit B	High expr		up					
nqrA	APL_0150	NADH:ubiquinone oxidoreductase, Na(+)-translocating, A subunit	High expr							STM
pflB	APL_1036	Formate acetyltransferase	High expr						down	
atpC	APL_1645	ATP synthase epsilon chain	High expr		up					
nqrC	APL_0152	Na(+)-translocating NADH-quinone reductase subunit C	High expr		up					
atpA	APL_1648	ATP synthase subunit alpha	High expr							STM
ackA	APL_0645	Acetate kinase	High expr			down				
nqrE	APL_0154	Na(+)-translocating NADH-quinone reductase subunit E	High expr		up					
APL_1591	APL_1591	Flavodoxin/protoporphyrinogen oxidase	Diff expr					HITS		
Cell cycle control, mitosis and meiosis										
fstE	APL_1345	Cell division ATP-binding protein	Diff expr					HITS		
ftsN	APL_1870	Possible cell division protein	Diff expr					HITS		
Amino acid transport and metabolism										
leuC	APL_0139	3-isopropylmalate dehydratase large subunit 2	Diff expr	up	down	up			up	
APL_0848	APL_0848	Putative ABC transporter periplasmic binding protein	Diff expr	up						
lysE	APL_1046	Lysine exporter protein	Diff expr		down					
pepA	APL_1101	Probable cytosol aminopeptidase	Diff expr						up	
thrC	APL_1499	Threonine synthase	Diff expr						up	STM
glnB	APL_1518	Nitrogen regulatory protein P-II	Diff expr					HITS		
ureA	APL_1618	Urease subunit gamma	Diff expr						up	
neuA	APL_1751	Acylneuraminate cytidylyltransferase	Diff expr	up						
proA	APL_1951	Gamma-glutamyl phosphate reductase	Diff expr			up				
ilvE	APL_0072	Branched-chain amino acid aminotransferase	Diff expr					HITS	up	
ilvC	APL_1853	Ketol-acid reductoisomerase	High expr						up	
Nucleotide transport and metabolism										

cpdB	APL_0646	2',3'-cyclic-nucleotide 2'-phosphodiesterase	Diff expr	down		down			
cdd	APL_1343	Cytidine deaminase	Diff expr					up	
prsA	APL_0775	Ribose-phosphate pyrophosphokinase	Diff expr	up		up			
Carbohydrate transport and metabolism									
glxK	APL_0142	Glycerat kinase	Diff expr					up	
glpF	APL_0374	Glycerol uptake facilitator protein	Diff expr		down				
glpT	APL_0377	Glycerol-3-phosphate transporter	Diff expr					up	
pgm	APL_0591	Phosphoglucomutase/phosphomannomutase	Diff expr				HITS		
APL_0707	APL_0707	Putative uncharacterized protein	Diff expr		up				
tktA	APL_0983	Transketolase 2	Diff expr			down		up	
malK	APL_1236	Maltose/maltodextrin import ATP-binding protein	Diff expr	up					
malG	APL_1239	Maltose transport system permease protein	Diff expr	up	up				
malQ	APL_1240	4-alpha-glucanotransferase	Diff expr	up					
mglA	APL_1419	Galactoside transport ATP-binding protein	Diff expr						STM
mgsA	APL_1498	Methylglyoxal synthase	Diff expr				HITS	down	
mtlA	APL_1630	PTS system mannitol-specific EIICBA component	Diff expr		down				
rbsB	APL_1672	D-ribose-binding periplasmic protein	Diff expr		down				
ulaD	APL_1698	Probable 3-keto-L-gulonate-6-phosphate decarboxylase	Diff expr	up					
nanE	APL_1752	Putative N-acetylmannosamine-6-phosphate 2-epimerase	Diff expr	up					
nagB	APL_1755	Glucosamine-6-phosphate deaminase	Diff expr	up			HITS		
rpe	APL_1820	Ribulose-phosphate 3-epimerase	Diff expr			up			
xylA	APL_1908	Xylose isomerase	Diff expr		down			up	
xylH	APL_1911	ABC-type xylose transport system, permease component	Diff expr		down				
fba	APL_1250	Fructose-bisphosphate aldolase	High expr			down			
gapA	APL_0434	Glyceraldehyde-3-phosphate dehydrogenase	High expr			down			
APL_0141	APL_0141	Uncharacterized transporter	Diff expr	up					
ulaC	APL_1699	Ascorbate-specific phosphotransferase enzyme IIA component	Diff expr	up					
ptsN	APL_0335	PTS system, nitrogen regulatory IIA-like protein	High expr		up				
Coenzyme transport and metabolism									
chuW	APL_1523	Coproporphyrinogen III oxidase	Diff expr		up				
hemH	APL_1937	Ferrochelatase	Diff expr				HITS		
hemX	APL_1008	Putative uroporphyrin-III C-methyltransferase	High expr	down					
serC	APL_0702	Phosphoserine aminotransferase	Diff expr					up	
rimK	APL_0484	Ribosomal protein S6 modification protein	Diff expr					up	
dxs	APL_0207	1-deoxy-D-xylulose-5-phosphate synthase	High expr					down	
Lipid transport and metabolism									
APL_0971	APL_0971	Putative acyl CoA thioester hydrolase	High expr					up	
plsX	APL_1385	Phosphate acyltransferase	High expr					down	
Translation									
rplQ	APL_1785	50S ribosomal protein L17	High expr		up				
rpsQ	APL_1769	30S ribosomal protein S17	High expr		up				
rpsJ	APL_1759	30S ribosomal protein S10	High expr	down					

rpsM	APL_1781	30S ribosomal protein S13	High expr						down	
rplI	APL_1169	50S ribosomal protein L9	High expr	down						
rplL	APL_1721	50S ribosomal protein L7/L12	High expr		up					
tufB	APL_1398	Elongation factor Tu	High expr				SCOTS			
rpsL	APL_0601	30S ribosomal protein S9	High expr		up					
rpsK	APL_1782	30S ribosomal protein S11	High expr	down						
rpmF	APL_1386	50S ribosomal protein L32	High expr							STM*
rpsA	APL_0740	30S ribosomal protein S1	High expr			down				
rplX	APL_1770	50S ribosomal protein L24	High expr		up					
Transcription										
nusA	APL_0638	Transcription elongation protein	Diff expr/high		up					
APL_0932	APL_0932	Putative HTH-type transcriptional regulator	High expr		down					
nusG	APL_1717	Transcription antitermination protein	High expr	down						
rho	APL_0247	Transcription termination factor	High expr		up					
Replication, recombination and repair										
recC	APL_1884	Exodeoxyribonuclease V gamma chain	Diff expr					HITS		
tagL	APL_1931	3-methyladenine-DNA glycosidase	Diff expr						up	
priB	APL_1170	Primosomal replication protein n	High expr	down						
deaD	APL_0575	Cold-shock DEAD box protein A-like protein	High expr		up	up				
Cell wall/membrane biogenesis										
murD	APL_0016	UDP-N-acetylmuramoylalanine--D-glutamate ligase	Diff expr			up				
ompW	APL_1086	Outer membrane protein W	Diff expr		up				down	
murI	APL_1841	Glutamate racemase	Diff expr			up				
mltC	APL_1741	Membrane-bound lytic murein transglycosylase C	High expr					HITS		
prc	APL_0120	Carboxy-terminal protease	High expr							STM
nlpC	APL_0359	Putative lipoprotein	High expr	down						
APL_1597	APL_1597	Possible rare lipoprotein A	High expr	down						
csgG	APL_0220	Putative lipoprotein	High expr	up					up	
ompA	APL_1421	Outer membrane protein P5 precursor	High expr				SCOTS			
ompA	APL_1852	Outer membrane protein P5 precursor (OMP P5)	High expr							STM*
Cell motility										
apfB/hofB	APL_0879	Fimbrial biogenesis protein	Diff expr	up						
Posttranslational modification, protein turnover, chaperones										
hypF	APL_1330	Hypothetical carbamoyltransferase	Diff expr			up				
tpx	APL_1489	Thiol peroxidase	Diff expr						up	
ureE	APL_1614	Urease accessory protein	Diff expr						up	
ptrA	APL_1883	Protease III	Diff expr						up	
dnaJ	APL_1905	Chaperone protein	High expr			up				STM
dnaK	APL_1906	Chaperone protein	High expr				SCOTS			STM*
ureG	APL_1612	Urease accessory protein	Diff expr						up	
Inorganic ion transport and metabolism										
nrfB	APL_0101	Cytochrome c-type protein	Diff expr		up	down				

nhaB	APL_0322	Na(+)/H(+) antiporter	Diff expr		up			HITS	
APL_0668	APL_0668	Possible periplasmic iron (Fe) transport lipoprotein	Diff expr	up	up				
ywbN	APL_0669	Tat-translocated enzyme, putative iron dependent peroxidase	Diff expr						down
kefBC	APL_1053	Glutathione-regulated potassium-efflux system protein	Diff expr				SCOTS		
tehA	APL_1212	Tellurite resistance protein and related permease	Diff expr						down
copA	APL_1265	Copper-transporting P-type ATPase	Diff expr				SCOTS		
APL_1285	APL_1285	Rhodanese-related sulfurtransferase	Diff expr						
ccp	APL_1379	Cytochrome c peroxidase	Diff expr		up	down			
APL_0719	APL_0719	Putative phosphate permease	High expr			down			
APL_1508	APL_1508	Rhodanese-related sulfurtransferase	High expr	down					
fur	APL_1218	Ferric uptake regulator	High expr	down					
hmuV/fecE	APL_1793	Hemin ABC superfamily ATP binding cassette transporter	Diff expr			up			
Secondary metabolites biosynthesis, transport and catabolism									
APL_1423	APL_1423	Putative uncharacterized protein	Diff expr						up
Signal transduction mechanisms									
typA	APL_0053	GTP-binding protein	High expr						down
Intracellular trafficking and secretion									
ftsY	APL_1346	Cell division protein FtsY-like protein	Diff expr						down
tatA	APL_1985	Sec-independent protein translocase protein	Diff expr					HITS	
secF	APL_1068	Protein-export membrane protein SecF	High expr						down
secB	APL_1509	Protein-export protein secB	High expr	down	up				
tatB	APL_1986	Sec-independent protein translocase-like protein	High expr					HITS	
Defence mechanisms									
apxID	APL_1442	RTX-I toxin secretion component	High expr						down
General function prediction only									
APL_0047	APL_0047	Diadenosine tetraphosphatase	Diff expr				SCOTS		
ompP4	APL_0389	Lipoprotein E	Diff expr			down		HITS	
APL_0885	APL_0885	Permease with 5 transmembrane domains	Diff expr		down				
APL_0966	APL_0966	Putative transport protein	Diff expr	up					
APL_1044	APL_1044	Permease	Diff expr	up					
dcuB2	APL_1316	Anaerobic C4-dicarboxylate transporter	Diff expr				SCOTS		
APL_1355	APL_1355	Phospholipid-binding protein	Diff expr						up
ulaG	APL_1701	L-ascorbate-6-phosphate lactonase	Diff expr	up					
trmB	APL_1383	tRNA (guanine-N(7)-)-methyltransferase	High expr		up				down
sspB	APL_0657	ClpXP protease specificity-enhancing factor	High expr	down					
nlpI	APL_0576	Lipoprotein NlpI-like	High expr	down	up				
Function unknown									
APL_0179	APL_0179	Putative uncharacterized protein	Diff expr						down
rimP	APL_0637	Ribosome maturation factor	Diff expr/high		up				
APL_1284	APL_1284	Putative DNA-binding protein	Diff expr			down			
APL_1360	APL_1360	Putative uncharacterized protein	Diff expr		up				
ulaA (sgaT)	APL_1700	PTS system ascorbate-specific transporter subunits IICB	Diff expr	up		up			

APL_1894	APL_1894	Putative uncharacterized protein	Diff expr		up
APL_0116	APL_0116	Putative DNA polymerase III subunit delta'	High expr	down	

Comparison of *A. pleuropneumoniae* , serotype 2 and 6, genes identified as differentially regulated or highly expressed in this study with:
I: *In vivo* infection with *A. pleuropneumoniae,* serotype 5b, (acute phase of natural infection) [11].
II: *A. pleuropneumoniae,* serotype 1, grown in BALF (30 min exposure) [21].
III: *A. pleuropneumoniae* grown on SJPL cells (for 3 h) [17].
IV: Genes expressed by *A. pleuropneumoniae,* serotype 9, in necrotic porcine lung tissue (7 days p.i.) [18].
V: Genes required by *H. influenzae* for survival in an experimental murine lung model [25].
VI: Genes regulated in *A. pleuropneumoniae,* serotype 1*,* during biofilm formation *in vitro* [22].
VII: Genes required for survival of *A. pleuropneumoniae* , serotype 1, in the porcine lung [15,17]. * Identified in both STM studies.
