# Supplementary material for: Transcriptional Portrait of Actinobacillus pleuropneumoniae during Acute Disease - Potential Strategies for Survival and Persistence in the Host
Source: PLoS One. 2012 Apr 17;7(4):e35549. doi: 10.1371/journal.pone.0035549 (PMC3328466; doi:10.1371/journal.pone.0035549)
Supplement: Table S6 — List of primers used for quantitative real-time PCR. (PDF) [file pone.0035549.s007.pdf]

Table S6. List of primers used for quantitative real-time PCR

Target gene	Forward primer	Reverse primer
glyA	5’-CAAGCGAATGCAGCTGTTTA-3’	5’-CTGTGATGCCGTAGAGGACA-3’
pykA	5’-GTACGGATGCGGTAATGCTT-3’	5’-GTACGGATGCGGTAATGCTT-3’
tpiA	5’-CTACGAACCGATTTGGGCTA-3’	5’-CCGCCGTATTGGATAATCAC-3’
tonB1	5’-CATTGCATTGCCATAACCAG-3’	5’-AAAAGCGCCTGAAAAGATCA-3’
tbpA1	5’-AGGAATGACGTTGGTTTTGC-3’	5’-ATTGCAGGTAGGGCTGATTG-3’
tonB2	5’-GCCTTGTACCGCATTAGGAA-3’	5’-CTCAGCCTAAGCCGAAAGAA-3’
hlyX	5’-TTTTACGTTGAGCGAACACG-3’	5’-ACGCCGTAATTTGTTCTTCG-3’
cirA	5’-TACGCTCTCCGGTGTGTATG-3’	5’-GTTGCGGTAGAAGCACCTTT-3’
ywbN	5’-TCGCAAATGGGCTTTAATTC-3’	5’-CTTTCAGCCAACCGTCTTGT-3’
lldD	5’-AATGCCCTTGATTACCATCG-3’	5’-GTAAACCGCATACGCTTGGT-3’
hybB	5’-TAATACCGGCAAAGGCTGTC-3’	5’-ACTTTCGCAAACTCGCCTAA-3’
fdxG	5’-TACTGTTCTGTCGGCTGTGG-3’	5’-GACTTACCGGATGGTCAGGA-3’
ykgE	5’-GTTTAAACGACCGGCAATGT-3’	5’-AACAAACCTGTTGCGGTTTC-3’
hyaA	5’-TTTACCGGGTATGCCGATTA-3’	5’-GTGTCCTTCATCGCCGTATT-3’
luxS	5’-CGTGTTGCAAAAACGATGAC-3’	5’-GCATAAAGCCGGCAAATAAG-3’
csrA	5’-CATCATCCGCTAATGCTTTG-3’	5’-ATTACCGTTCTCAGCGTTCG-3’
manB	5’-AGAAGAAGCAACGGTCGAAA-3’	5’-ACCCGATTCTGCATGAAAAC-3’
yfhL	5’-ACAGTTGGTAATCGGGCAAA-3’	5’-AATCGGCGATGATGTGTATG-3’
nusA	5’-AAACGCAATTACCGCAAAAG-3’	5’-TTCTTCAAGCGTTGTGATGC-3’
apxIIA	5’-GAATCATTTCCATCGCCTGT-3’	5’-TCGATGGAGGAAACGGTAAC-3’
apfB	5’-CGGAAGACGGGATTCACTAA-3’	5’-GGAACCGCAAAAAGACAAAA-3’
nrfG	5’-TGCTCTCGCCGAAATCTTAT-3’	5’-CGCATTGGTTTGTTACGACA-3’
glpQ	5’-TCCGATTTTTGGTCATAGCC-3’	5’-CGGTAAACAAATCGGCATCT-3’
rraA	5’-AACACGCTGGCAATTAAACC-3’	5’-GTCGAGCCAATCTTTTCGAG-3’
kdsB	5’-TGAAACAATACGTCGCTTGG-3’	5’-GCGAACACGTTCCAAATCTT-3’
yegQ	5’-TCAAGGCACTTGCACTAACG-3’	5’-TTCGATTTCCGGTTCATAGC-3’
ftsY	5’-AAATTACGGAAACCGGAACC-3’	5’-CGAGCTTGAAACGGAAAAAG-3’
ompP4	5’-TGCGCAAGTGCTTGATATTC-3’	5’-GCTCATCACGATACGAGCAA-3’
hgbA	5’-ATAAGGTCTTGCCGTTGCAC-3’	5’-AGACGAAAGAAGCCGCATTA-3’
wecD	5’-GAATCAGCTTGCGTTTAGCC-3’	5’-AGATTCATCGCAGGCAAGTT-3’
wecE	5’-GCCGTTTGGCAACACTATTT-3’	5’-AACAGATGGCCGTTTTGTTC-3’
nanA	5’-GTAAATTGGCGGAAGCGTTA-3’	5’-CGGCTTCCACACCTTGTAAT-3’
neuA	5’-GAGCAAATTAGACCGCTTGC-3’	5’-AATGTAATATGGGGCGGACA-3’
nagB	5’-TGCCGAAATACGCTTTAACC-3’	5’-TATGGTTTACCGAGCCTTCG-3’
