# Supplementary material for: Transcriptional Portrait of Actinobacillus pleuropneumoniae during Acute Disease - Potential Strategies for Survival and Persistence in the Host
Source: PLoS One. 2012 Apr 17;7(4):e35549. doi: 10.1371/journal.pone.0035549 (PMC3328466; doi:10.1371/journal.pone.0035549)
Supplement: Table S7 — R2 values and Efficiency of standard curves used in qPCR analyses. (PDF) [file pone.0035549.s008.pdf]

Table S7. R^2 values and Efficiency of standard curves used in qPCR analyses

**Target gene**	**Annotation**	$\mathbf{R^2}$	**Efficiency**
Primers used to quantify amplified versus non-amplified mRNA			
glyA*	Serine hydroxymethyltransferase	1.0	0.89
pykA*	Pyruvate kinase	1.0	0.93
tpiA	Triosephosphate isomerase	1.0	0.96
luxS	S-ribosylhomocysteinase	1.0	0.86
cirA	ABC transporter ATP-binding protein	1.0	0.81
hybB	Hydrogenase 2 b cytochrome subunit	1.0	0.92
fdxG	Formate dehydrogenase	1.0	1.00
tonB1	Periplasmic protein	1.0	0.97
tonB2	TonB2 protein	1.0	1.01
tbpA1	Transferrin-binding protein 1	1.0	0.92
hlyX	Fumarate/nitrate reduction transcriptional regulator	1.0	0.90
hyaA	Hydrogenase 2 small subunit	1.0	1.07
lldD	L-lactate dehydrogenase	1.0	0.96
Primers used to validate microarray data			
csrA*	Carbon storage regulator	1.0	1.01
manB*	Phosphomannomutase	1.0	1.02
yfhL	Ferredoxin-like protein	1.0	1.00
nusA	Transcription elongation factor	1.0	1.01
ywbN	Putative iron dependent peroxidase	1.0	1.08
hybB	Putative hydrogenase 2 b cytochrome subunit	1.0	1.04
ykgE	Putative dehydrogenase subunit	1.0	0.92
apxIIA	RTX-II toxin determinant A	1.0	0.90
apfB	Fimbrial biogenesis protein	1.0	1.00
nrfG	Formate-dependent nitrite reductase complex subunit	1.0	0.90
glpQ	Glycerophosphodiester phosphodiesterase	1.0	1.00
rraA	Ribonuclease activity regulator protein	1.0	1.02
kdsB	Deoxy-manno-octulosonate cytidylyltransferase	1.0	1.02
yegQ	Uncharacterized protease	1.0	1.00
ftsY	Cell division protein	1.0	1.00
ompP4	Lipoprotein E	1.0	0.90
hgbA	Hemoglobin binding protein A	1.0	1.02
wecD	Putative TDP-D-fucosamine acetyltransferase	1.0	0.94
wecE	TDP-4-keto-6-deoxy-D-glucose transaminase	1.0	1.06
nanA	N-acetylneuraminate lyase	1.0	0.84
neuA	Acylneuraminate cytidylyltransferase	1.0	0.90
nagB	Glucosamine-6-phosphate deaminase	1.0	0.90

*Reference genes applied for data normalization.
